# Supplementary material for: MicroRNA-125b Promotes Hepatic Stellate Cell Activation and Liver Fibrosis by Activating RhoA Signaling
Source: Mol Ther Nucleic Acids. 2018 May 3;12:57–66. doi: 10.1016/j.omtn.2018.04.016 (PMC6023793; doi:10.1016/j.omtn.2018.04.016)
Supplement: Document S1. Figures S1–S10 and Tables S1 and S2 [file mmc1.pdf]

OMTN, Volume *12*

Supplemental Information

MicroRNA-125b Promotes Hepatic Stellate Cell Activation and Liver Fibrosis by Activating RhoA Signaling

Kai You, Song-Yang Li, Jiao Gong, Jian-Hong Fang, Chong Zhang, Min Zhang, Yunfei Yuan, Jine Yang, and Shi-Mei Zhuang

Supplemental Data:

Supplemental Figures

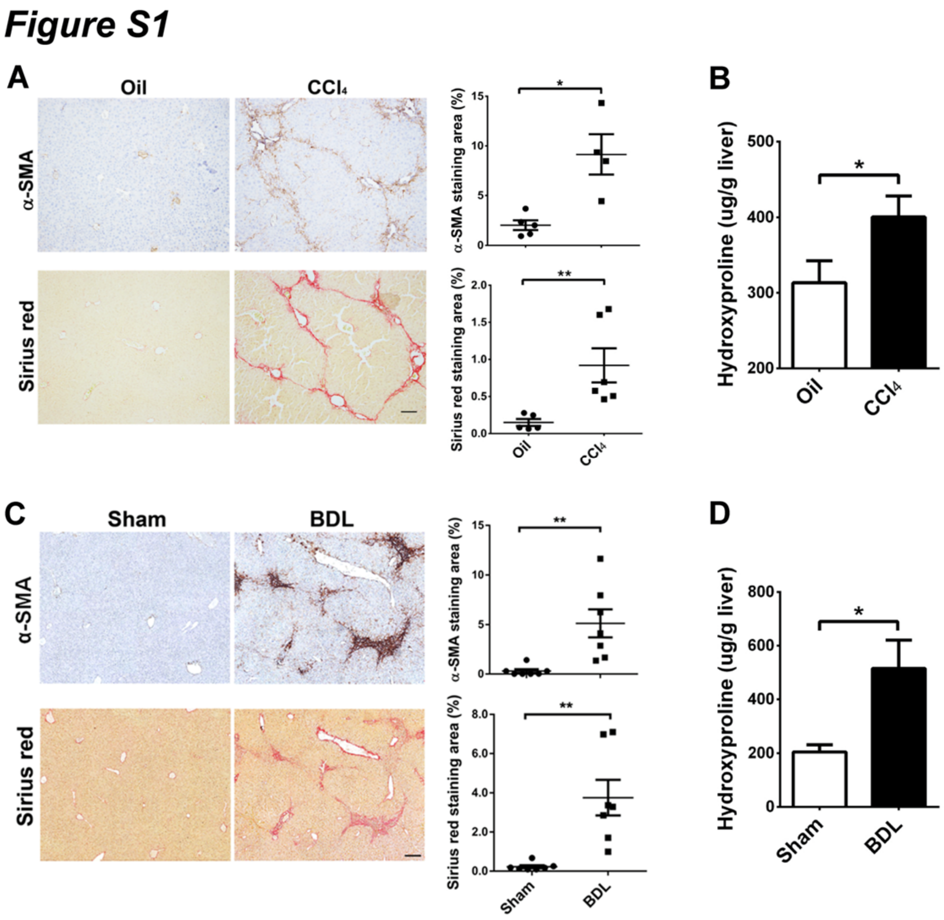

Figure S1. Induction of liver fibrosis in mice. (A-B) CCl_4 induced liver fibrosis in mice. Male C57BL/6 mice at 6 weeks of age were treated with CCl_4 (0.5 μL/g body weight) for 6 weeks. (C-D) BDL induced liver fibrosis in mice. Male C57BL/6 mice at 6 weeks of age were treated with bile duct ligation for 3 weeks. For (A) and (C), liver fibrosis was assessed by α-SMA and Sirius red staining (magnification, ×100). Percentage of positive staining area relative to total area was quantified by image pro plus version 6.0 software and shown in the *right*. For (B) and (D), hydroxyproline content of the livers was determined by Hydroxyproline Colorimetric Assay kit. Scale bar, 100 μm. Data are presented as mean± SEM in (A)-(D). *, $P < 0.05$; **, $P < 0.01$.

Figure S2

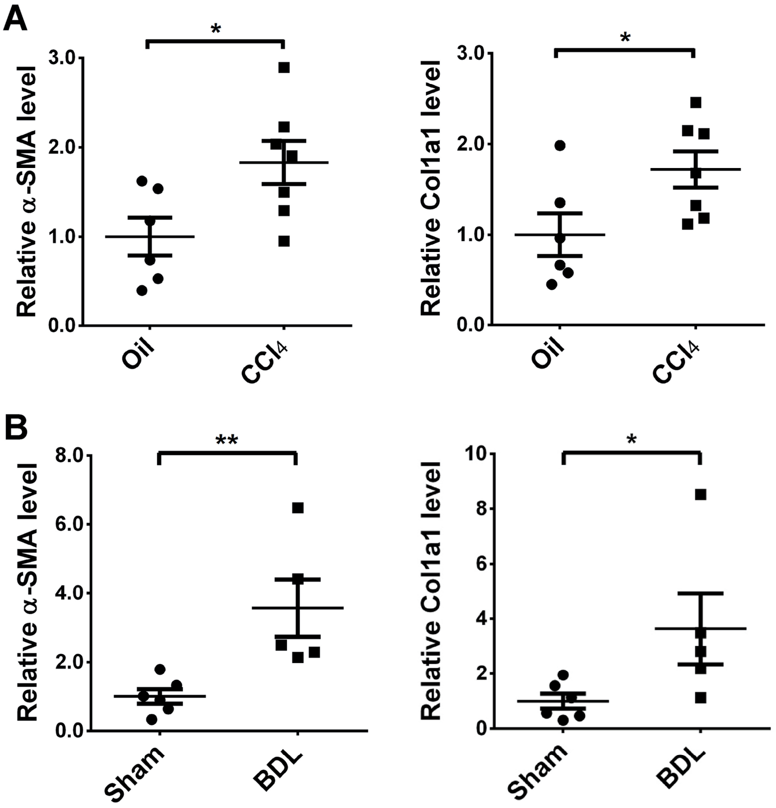

Figure S2. Expression of *α-SMA* and *Col1a1* was increased in the HSCs of fibrotic livers. HSCs were isolated from the fibrotic livers of mice treated with CCl_4 (A) or BDL (B) or from the normal livers of control mice, and then cultured for 24 hours before qRT-PCR analysis. The mean expression level of each gene in control group was set as relative level 1. Data are presented as mean ± SEM in (A)-(B). *, $P < 0.05$; **, $P < 0.01$.

Figure S3

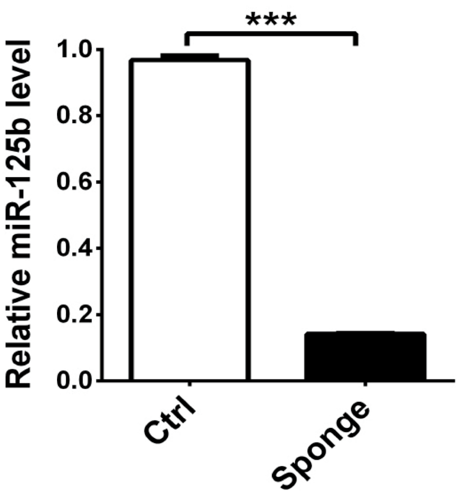

Figure S3. The level of miR-125b was decreased in the JS1 cells infected with miR-125b sponge-lentivirus. JS1 cells were infected by control (Ctrl) or miR-125b-sponge-lentivirus (Sponge) to establish stable infected cell lines. In passage 4, the expression level of miR-125b in both Ctrl and Sponge groups was analyzed by qRT-PCR. Data are presented as mean ± SEM. *** $P < 0.001$.

Figure S4

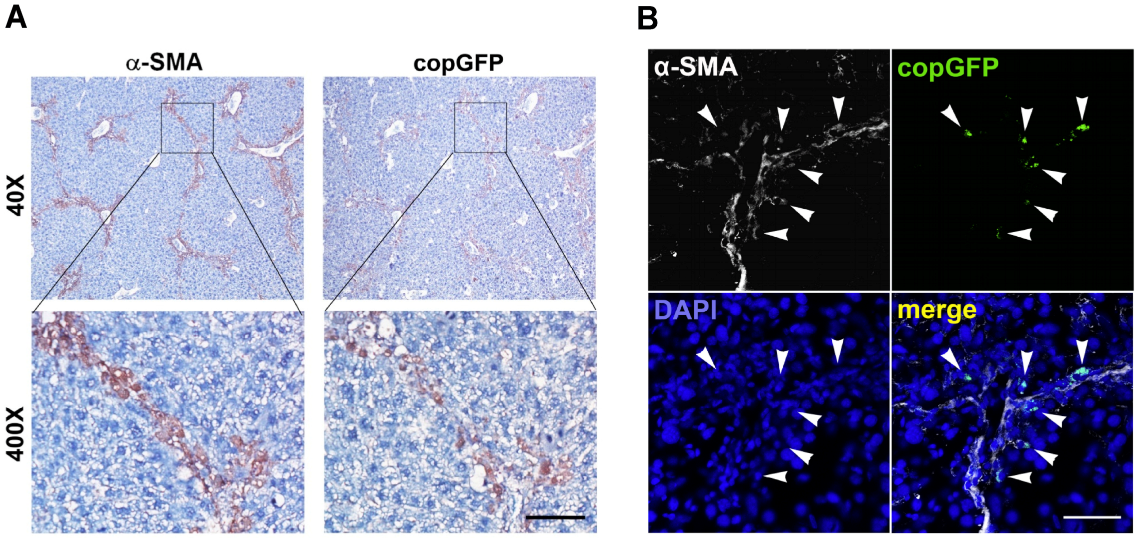

Figure S4. Activated HSCs were infected by lentiviruses *in vivo*. (A) Immunohistochemistry analysis was performed to determine the distribution of activated HSCs and the lentivirus-infected cells. Serial sections from the liver of CCl_4-treated mice, which were intravenously injected with lentiviruses, were stained with α-SMA and copGFP antibody, respectively. (B) Immunofluorescent staining was performed to determine the distribution of activated HSCs and the lentivirus-infected cells. Frozen liver sections were stained with α-SMA (white) and DAPI (blue). Lentivirus-infected cells showed green due to expression of copGFP. Arrowheads indicate the infection of lentiviruses in activated HSCs (co-localization of α-SMA and copGFP). Scale bar, 50 μm.

Figure S5

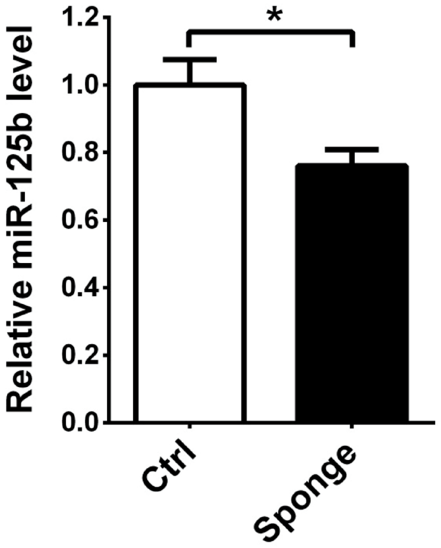

Figure S5. Administration of miR-125b-sponge reduced endogenous miR-125b level in mouse liver. miR-125b-sponge (Sponge) or its control (Ctrl) lentiviruses were injected through tail vein on the 5th and 7th day after the first administration of CCl_4. Mouse livers were collected and subjected to qRT-PCR analysis at 3 weeks after the last injection of lentiviruses. Data are presented as mean ± SEM. *, $P < 0.05$.

Figure S6

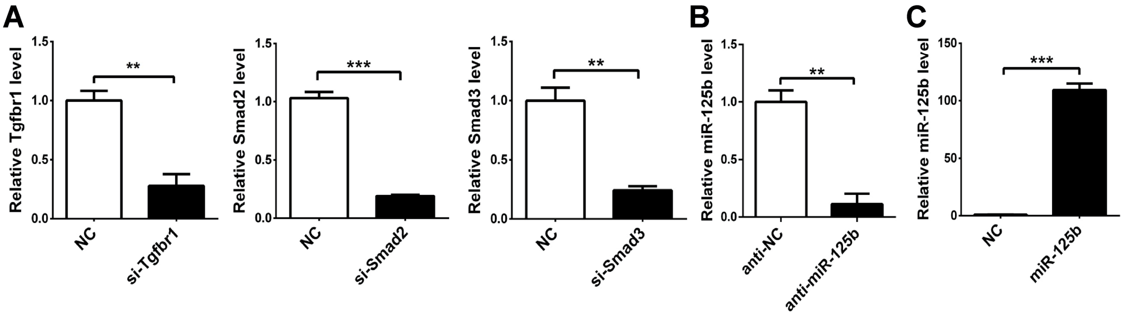

Figure S6. The transfection efficiency of RNA oligoribonucleotides. JS1 cells were transfected with the indicated RNA for 48 hours before qRT-PCR analysis. Data are presented as mean±SEM in (A)-(C). **, $P < 0.05$; ***, $P < 0.001$.

Figure S7

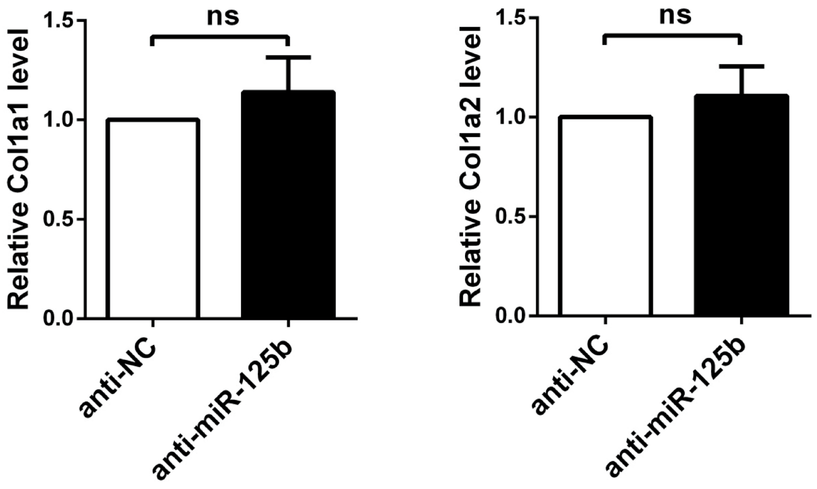

Figure S7. Knockdown of miR-125b did not affect the basal levels of *Col1a1* and *Col1a2*. JS1 cells were transfected with anti-NC or anti-miR-125b for 48 hours, then the mRNA levels of *Col1a1* and *Col1a2* were analyzed by qRT-PCR. Data are presented as mean±SEM. ns, not significant.

Figure S8

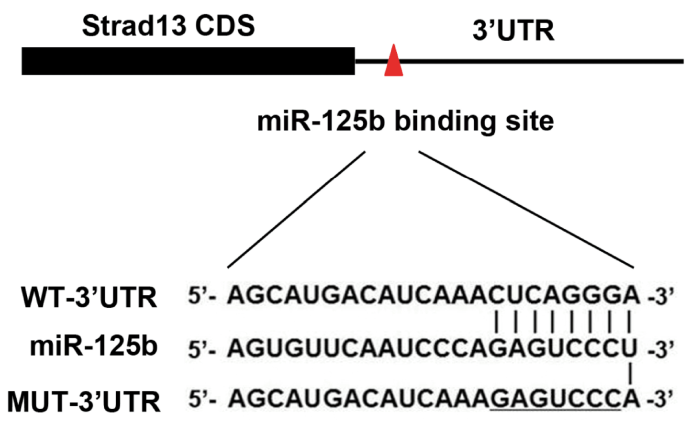

Figure S8. The schematic diagram of the luciferase reporter plasmids that contain either wild-type or mutant 3’UTR of *Stard13*. The segments containing miR-125b binding site were cloned into psiCHECK-2 vector.

Figure S9

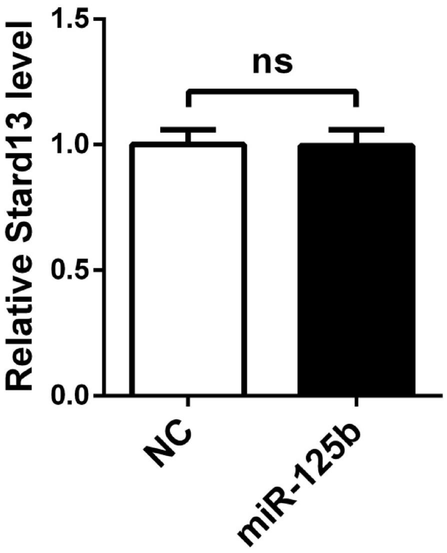

Figure S9. Overexpression of miR-125b did not affect the mRNA level of *Stard13*. JS1 cells were transfected with NC or miR-125b mimics for 48 hours, then the mRNA level of *Stard13* was analyzed by qRT-PCR. Data are presented as mean ± SEM. ns, not significant.

Figure S10

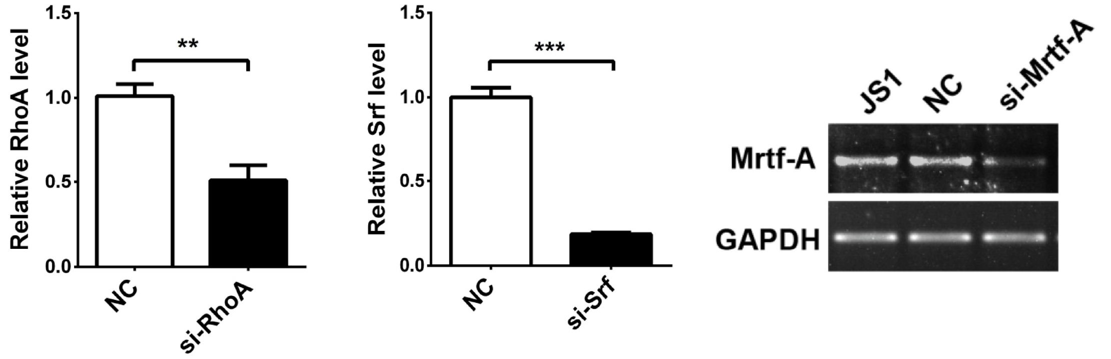

Figure S10: The knockdown effect of siRNAs for *RhoA, Srf* and *Mrtf-A*. JS1 cells were transfected with the indicated RNA for 48 hours and then subjected to qRT-PCR or RT-PCR analysis. Data are presented as mean±SEM. **, *P < 0.0*5; ***, *P < 0.0*01.

Supplemental Tables

Table S1. Characteristics of study subjects

Case	Gender	Age(years)	ALT(U/L)[1]	AST(U/L)[1]	TBIL(μmol/L)[1]	HBsAg[2]	Cirrhosis[3]
**Liver fibrosis**							
1	Male	60	741	1590	19.1	+	+
2	Male	36	39	29	10.8	+	+
3	Male	57	51	60	27.9	+	+
4	Male	60	46	45	20.9	+	+
5	Male	40	39	62	17	+	+
6	Male	61	163	104	770.1	+	+
7	Male	41	48	26	61	+	+
8	Male	38	19	10	48.4	-	+
9	Male	46	69	36	71.5	+	+
**Hepatic hemangiomas**							
1	Male	50	16.6	17.8	19	+	-
2	Female	56	11.9	16	12.7	-	-
3	Male	51	39.3	73.1	16.1	+	-
4	Female	42	34	44.6	9.6	+	-
5	Female	50	14.9	17.1	10.3	+	-
6	Female	53	16.2	16.4	10.8	-	-
7	Male	41	46	28.9	15.8	-	-
8	Male	49	17	19.3	13.3	-	-
9	Female	46	11.5	13.3	3.6	+	-

[1] ALT, alanine aminotransferase; AST, aspartate aminotransferase; TBIL, total bilirubin;

[2] HBsAg, hepatitis B surface antigen; detected by ELISA; "+", presence; "-", absence.

[3] Determined by pathological examination; "+", presence; "-", absence.

Table S2. Sequences of RNA and DNA Oligonucleotides

Name	Sense Strand (5'-3')	Antisense Strand (5'-3')
**miRNA and siRNA duplexes**		
miR-125b	UCCCUGAGACCCUAACUUGUGA	ACAAGUUAGGGUCUCAGGGCUU
si-Stard13-1	UGCUCUAUUCACAGCGAAAdTdT	UUUCGCUGUGAAUAGAGCAdGdA
si-Stard13-2	CAGACCAAACAGACGACUAdTdT	UAGUCGUCUGUUUGGUCUGdGdT
si-Stard13-3	GCUCCUGACUACCGAGACAdTdT	UGUCUCGGUAGUCAGGAGCdTdT
si-RhoA-1	GUGAUGGAGCUUGUGGUAAdTdT	UUACCACAAGCUCCAUCACdCdA
si-RhoA-2	CGGAAUGACGAGCACACGAdTdT	UCGUGUGCUCGUCAUUCCGdAdA
si-Srf-1	CCAACGGGACUGUGCUCAAdTdT	UUGAGCACAGUCCCGUUGGdCdA
si-Srf-2	CCUACCAGGUGUCGGAAUCdTdT	GAUUCCGACACCUGGUAGGdTdG
si-Tgfbr1-1	ACAAGAAAGCAUUGGCAAAdTdT	UUUGCCAAUGCUUUCUUGUdAdA
si-Tgfbr1-2	CUGACAGCUUUGCGAAUUAdTdT	UAAUUCGCAAAGCUGUCAGdCdC
si-Mrtf-A-1	CAAGUCUGCCAGCGAGAAAdTdT	UUUCUCGCUGGCAGACUUGdGdG
si-Mrtf-A-2	AGGACUAUUUGAAACGGAAdTdT	UUCCGUUUCAAAUAGUCCUdCdG
si-Smad2-1	GCUGAGUGCCUAAGUGAUAdTdT	UAUCACUUAGGCACUCAGCdAdA
si-Smad2-2	CAUCAUGAGCUCAAGGCAAdTdT	UUGCCUUGAGCUCAUGAUGdAdC
si-Smad3-1	CCGUAUGAGCUUCGUCAAAdTdT	UUUGACGAAGCUCAUACGGdAdT
si-Smad3-2	UGUCCAAUGUCAACCGGAAdTdT	UUCCGGUUGACAUUGGACAdGdT
NC	UUGUACUACACAAAAGUACUG	GUACUUUUGUGUAGUACAGUU
**miRNA inhibitors**		
anti-NC	GUGGAUAUUGUUGCCAUCA	
anti-miR-125b	UCACAAGUUAGGGUCUCAGGGA	
**Primers for RT-PCR or qRT-PCR**		
α-SMA	ATGCAGAAGGAGATCACAGC	CAGCTTCGTCGTATTCCTGT
Col1a1	ATGATGCTAACGTGGTTCGT	TGGTTAGGGTCGATCCAGTA
Col1a2	GGAGGGAACGGTCCACGAT	GAGTCCGCGTATCCACAA
Timp-1	GCAACTCGGACCTGGTCATAA	CGGCCCGTGATGAGAAACT
Fn1	ATGAGCGCCCTAAAGATTCC	CCAGCAGCATGATCAAAACA
Stard13	CCACTGGGGTCAGAGAAAGA	ATGATGGCCGTAAGACGAAG
RhoA	CTTCAGCAAGGACCAGTTCCCA	GGCGGTCATAATCTTCCTGTCC
Srf	ATGAGTGCCACTGGCTTTG	CTGTGAATGCTGGCTTCAGT
Tgfbr1	GGTCTTGCCCATCTTCACAT	TGGCAGAATCATGTCTCACAG
Mrtf-A	CCAGGACCGAGGACTATTTG	TGTGCAATCTTTTCATTGAGG
Smad2	ACTACACCCACTCCATTCCA	GCGATTGAACACCAGAATGC
Smad3	TTTTCGTCCAGTCTCCCAAC	CTGGTCACTGTCTGTCTCCT
GAPDH	AACTTTGGCATTGTGGAAGG	CACATTGGGGGTAGGAACAC
**Primers for cloning (Restriction enzyme sites were underlined)**		
Stard13-3'UTR-WT	AGTCTCGAGGCTCAGGACCTGGAAGACTG	AGTGCGGCCGCGGAAGTGCCATGTGAGGAGA
Stard13-3'UTR-MUT	CAGCATGACATCAAAGAGTCCCAAGAAGAAGGGAAAG	CTTTCCCTTCTTCTTGGGACTCTTTGATGTCATGCTG
pCDH-Ctrl	AGTGAATTCATGGTGAGCAAGGGCGAGGAG	AGTGGATCCTTACTTGTACAGCTCGTCCATGCC
pCDH-miR-125b sponge	AGTGAATTCATGGTGAGCAAGGGCGAGGAG	AGTGGATCCAGAGTGGAGTCGTCGACGCC
