# Supplementary material for: MicroRNA-125b Promotes Hepatic Stellate Cell Activation and Liver Fibrosis by Activating RhoA Signaling
Source: Mol Ther Nucleic Acids. 2018 May 3;12:57–66. doi: 10.1016/j.omtn.2018.04.016 (PMC6023793; doi:10.1016/j.omtn.2018.04.016)
Supplement: Document S2. Article plus Supplemental Information [file mmc2.pdf]

Molecular Therapy
Nucleic Acids
Original Article

MicroRNA-125b Promotes Hepatic Stellate Cell Activation and Liver Fibrosis by Activating RhoA Signaling

Kai You,[1] Song-Yang Li,[1] Jiao Gong,[2] Jian-Hong Fang,[1] Chong Zhang,[1] Min Zhang,[2] Yunfei Yuan,[3] Jine Yang,[1] and Shi-Mei Zhuang[1,2]

[1]Key Laboratory of Gene Engineering of the Ministry of Education, State Key Laboratory of Biocontrol, Collaborative Innovation Center for Cancer Medicine, School of Life Sciences, Sun Yat-sen University, Guangzhou, China; [2]Key Laboratory of Liver Disease of Guangdong Province, The Third Affiliated Hospital, Sun Yat-sen University, Guangzhou, China; [3]Cancer Center, Sun Yat-sen University, Guangzhou, China

miR-125b is frequently dysregulated in different diseases. Activation of hepatic stellate cells (HSCs) is a critical event during liver fibrogenesis. However, the function and its underlying mechanism of miR-125b in HSC activation and liver fibrosis are still unknown. Here, we showed that miR-125b was upregulated in HSCs, but not in hepatocytes, during hepatic fibrogenesis *in vivo* and upon culture activation *in vitro*. Inhibition of miR-125b suppressed the expression of profibrogenic genes in culture-activated primary HSCs and reduced the basal and transforming growth factor β (TGF-β)-induced alpha-smooth muscle actin (α-SMA) expression and cell contraction of the immortalized HSC cell line. In contrast, ectopic expression of miR-125b promoted α-SMA expression and HSC contraction. Moreover, antagonizing miR-125b *in vivo* significantly alleviated liver fibrosis in CCl_4-treated mice. Mechanistically, overexpression of miR-125b in HSCs enhanced RhoA activity by directly targeting StAR-related lipid transfer (START) domain containing 13 (Stard13), a RhoA-specific GTPase-activating protein, whereas knockdown of miR-125b abrogated RhoA activation. Furthermore, inhibition of RhoA or its downstream molecules, Mrtf-A and Srf, attenuated the miR-125b-induced α-SMA expression and HSC contraction. Therefore, our findings identify a miR-125b-Stard13-RhoA-α-SMA signaling cascade in HSCs and highlight its importance in hepatic fibrosis.

INTRODUCTION

Hepatic fibrosis is the outcome of chronic liver injury in response to various etiologies. It is characterized by excess accumulation of abnormal extracellular matrix (ECM) and disruption of hepatic architecture.[1,2] During liver fibrosis, ECM is converted from a collagen IV/VI-rich matrix to a collagen I/III-rich one, resulting in liver stiffness and distortion of the sinusoidal architecture. Long-term fibrosis leads to cirrhosis and subsequent liver dysfunction and hepatocellular carcinoma (HCC).[3] Activation of hepatic stellate cells (HSCs) plays a critical role in liver fibrosis and is characterized by the conversion from vitamin A-storing cell into myofibroblast that expresses excess profibrogenic genes, such as alpha-smooth muscle actin (*α-SMA*), collagen type I alpha 1 chain (*Col1a1*), collagen type I alpha 2 chain (*Col1a2*), tissue inhibitor of metalloproteinase 1 (*Timp1*), and fibronectin 1 (*Fn1*). α-SMA is a marker for activated HSCs, and it also enhances the contraction force of HSCs by incorporating into stress fibers.[4,5] The contractility of HSCs leads to increased liver stiffness and hepatic sinusoidal vasoconstriction, two key events that cause liver complications, like portal hypertension.[6,7] Identification of molecules that regulate HSC activation and liver fibrogenesis may provide novel therapeutic targets. To date, whether microRNAs (miRNAs) have a role in these processes remains poorly understood.

miR-125b is frequently dysregulated in human diseases, especially in cancers. Recent reports have shown that miR-125b is also implicated in different liver diseases. For instance, miR-125b attenuates paracetamol- and FAS-induced toxicity in hepatocytes and prevents acute liver failure (ALF) by directly targeting kelch-like ECH-associated protein 1 (*Keap1*);[8] upregulation of miR-125b protects female mice from non-alcoholic fatty liver disease through repressing lipid accumulation in hepatocytes,[9] and downregulation of miR-125b in cholangiocyte enhances proliferation of cholangiocytes during bile duct ligation (BDL)-induced cholestatic liver injury.[10] In addition, miR-125b promotes apoptosis and inhibits proliferation and metastasis of HCC cells.[11,12] Some studies have also revealed that miR-125b level is increased in mouse fibrotic livers[13] and in the serum of patients with liver fibrosis.[14] However, the function and its underlying mechanism of miR-125b in HSC activation and liver fibrosis remain unknown.

Received 29 April 2018; accepted 29 April 2018;
https://doi.org/10.1016/j.omtn.2018.04.016.

Correspondence: Shi-Mei Zhuang, School of Life Sciences, Sun Yat-sen University, 135 Xin Gang Xi Road, Guangzhou 510275, China.
E-mail: zhuangshimei@163.com
Correspondence: Jine Yang, School of Life Sciences, Sun Yat-sen University, 135 Xin Gang Xi Road, Guangzhou 510275, China.
E-mail: lssyje@mail.sysu.edu.cn

This is an open access article under the CC BY license (http://creativecommons.org/licenses/by/4.0/).

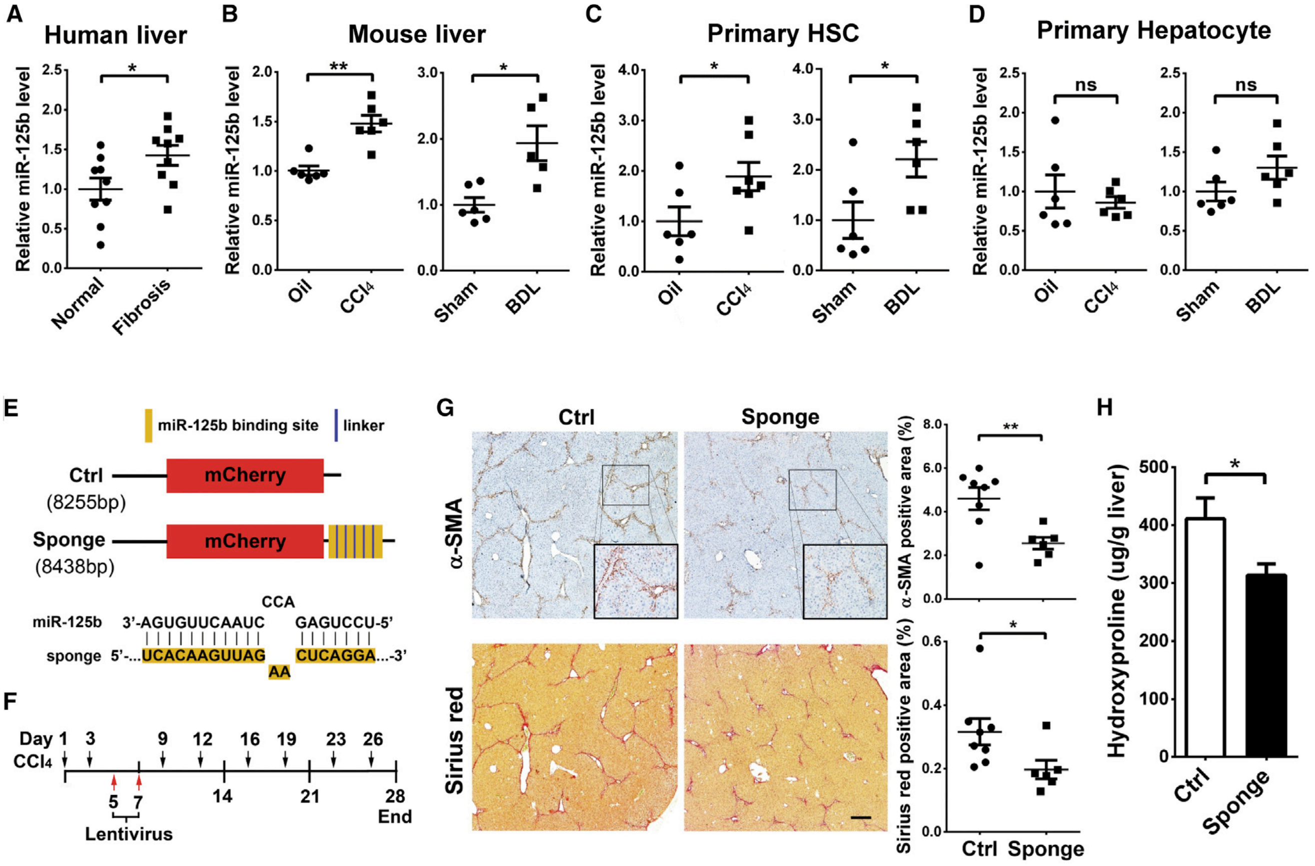

Figure 1. miR-125b Is Increased in HSCs of Fibrotic Livers, and Inhibition of miR-125b Attenuates Hepatic Fibrosis *In Vivo*

(A and B) The level of miR-125b was increased in fibrotic livers. Samples were collected from human (A) and CCl_4- or BDL-treated mice (B). (C and D) miR-125b was upregulated in the activated HSCs, but not in hepatocytes, isolated from the fibrotic livers of CCl_4- or BDL-treated mice. Mouse primary HSCs (C) and hepatocytes (D) were isolated from normal or fibrotic livers. (A–D) miR-125b was detected by qRT-PCR analysis, and the mean value of miR-125b expression in the control group was set to 1. (E) Schematic diagram of the lentiviral vector of miR-125b-sponge and its control. Control lentivirus (Ctrl) carried *mCherry* reporter gene driven by the cytomegalovirus (CMV) promoter. miR-125b-sponge (sponge) had the same structure as the control lentivirus except that six tandem repeats of the miR-125b binding sequence were inserted immediately after the stop codon of the mCherry coding sequence. The consensus sequence of the miR-125b binding site in the sponge is shown. (F) An illustration showing experimental procedures of antagonizing endogenous miR-125b with lentiviral sponge in CCl_4-treated mice. Liver fibrosis was induced in mice by an intraperitoneal injection of CCl_4 twice weekly for 4 weeks. Lentivirus with the miR-125b-sponge or control lentivirus was injected through the tail vein on the fifth and seventh days after the first administration of CCl_4. Mouse livers were collected and subjected to immunohistochemistry analysis or hydroxyproline assay 3 weeks after the last injection of lentiviruses. (G and H) Antagonizing endogenous miR-125b alleviated liver fibrosis *in vivo*. Expression of α-SMA and deposition of collagen were detected by immunohistochemistry staining and Sirius Red staining (G), respectively. Hydroxyproline content of livers was determined by the Hydroxyproline Colorimetric Assay kit (H). Scale bar, 250 μm. Data are presented as mean ± SEM in (A)–(D), (G), and (H). *p < 0.05; **p < 0.01. ns, not significant.

Here, we showed that miR-125b was significantly upregulated in murine activated HSCs, but not in hepatocytes, during liver fibrogenesis. Inhibition of miR-125b dramatically suppressed the activation of murine HSCs *in vitro* and attenuated hepatic fibrosis *in vivo*. Moreover, miR-125b enhanced the activity of RhoA by directly targeting StAR-related lipid transfer (START) domain containing 13 (*Stard13*) expression, consequently increasing the level of α-SMA and the contractile ability of HSCs. This study reveals a promotive function of miR-125b in HSC activation and hepatic fibrosis, and this may be beneficial for developing miR-125b-based therapy for liver fibrosis.

RESULTS

miR-125b Is Upregulated in Activated HSCs, and Inhibition of miR-125b Attenuates Hepatic Fibrosis *In Vivo*

To assess the function of miR-125b in liver fibrosis, we first evaluated by qRT-PCR the expression of miR-125b in fibrotic liver tissues. Compared with normal livers, the level of miR-125b was significantly increased in human fibrotic livers (Figure 1A), and also in mouse fibrotic livers (Figure 1B) that were derived from the mice with treatment of carbo tetrachloride (CCl_4) or BDL (Figures S1A–S1D), two widely used models of experimental liver fibrosis. To clarify which cell type displayed miR-125b

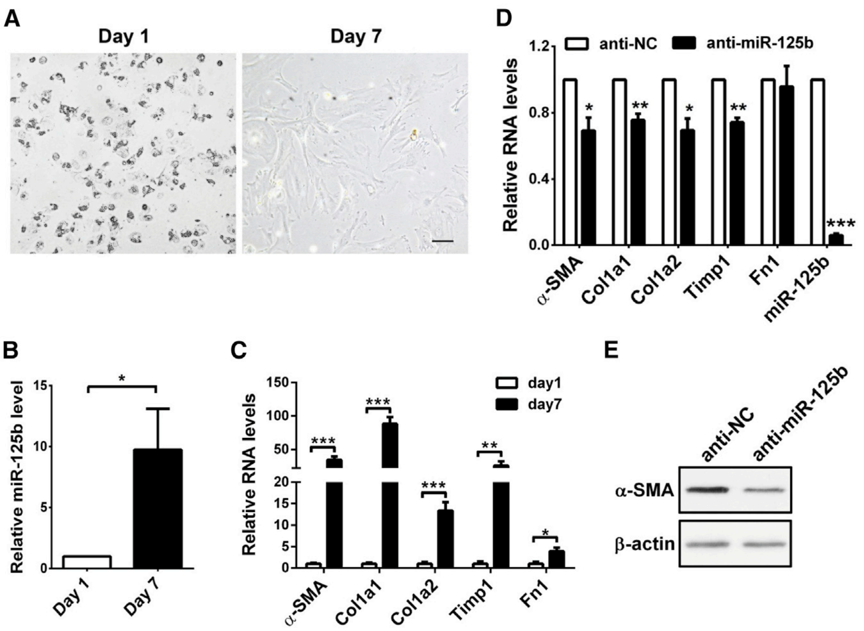

Figure 2. Inhibition of miR-125b Attenuates the Activation of Primary HSCs *In Vitro*

(A) Phase-contrast images showing the morphological changes of culture-activated HSCs. Mouse primary HSCs were isolated from normal livers and then cultured for the indicated times before capturing the images. Scale bar, 100 μm. (B) miR-125b was upregulated in culture-activated HSCs. (C) Expression levels of *α-SMA*, *Col1a1*, *Col1a2*, *Timp1*, and *Fn1* were increased in culture-activated HSCs. For (B) and (C), mouse primary HSCs that were cultured in a 12-well plate for 1 or 7 days were subjected to qRT-PCR analysis. (D and E) Inhibition of cellular miR-125b reduced the expression of *α-SMA*, *Col1a1*, *Col1a2*, and *Timp1* in culture-activated HSCs. Mouse primary HSCs were cultured in a 12-well plate for 4 days, then transfected with anti-NC (negative control) or anti-miR-125b and incubated for 72 hr before qRT-PCR (D) or western blotting (E) analysis. In (D), the level of each gene in anti-NC transfectants was set as relative level 1. Data are presented as mean ± SEM in (B)–(D). $^*p < 0.05$; $^{**}p < 0.01$; $^{***}p < 0.001$

dysregulation during fibrosis, we analyzed miR-125b level in primary hepatocytes and HSCs isolated from murine livers. Compared with primary HSCs from normal livers, those from the fibrotic livers of CCl_4- or BDL-treated mice showed a significant upregulation in miR-125b (Figure 1C), *α-SMA*, and type I collagen (Figures S2A and S2B) expression. However, hepatocytes from fibrotic and normal livers exhibited similar miR-125b levels (Figure 1D). These data suggest that increase of miR-125b in HSCs may contribute to liver fibrosis.

We then explored whether antagonism of miR-125b could block liver fibrosis *in vivo*, using "sponge" strategy[15] to sequester cellular endogenous miR-125b. The miR-125b-sponge-lentivirus, which contained six tandemly repeated miR-125b binding sites after the stop codon of mCherry coding gene and expressed copGFP, was generated (Figure 1E), and its role in reducing miR-125b level was verified in an immortalized HSC cell line, JS1 (Figure S3). The miR-125b-sponge-lentivirus and its control were then intravenously injected into CCl_4-treated mice at days 5 and 7 after the first administration of CCl_4 (Figure 1F). Both immunohistochemical and immunofluorescent analyses showed co-localization of copGFP and α-SMA staining in the fibrous septa area (Figures S4A and S4B), indicating infection of lentiviruses in activated HSCs. Furthermore, compared with the livers from the control group, those from the miR-125b-sponge group displayed a lower level of miR-125b (Figure S5) and much less HSC activation and collagen deposition, as evidenced by reduced α-SMA and Sirius red staining (Figure 1G), and decreased hydroxyproline content in the livers (Figure 1H), suggesting an alleviation of hepatic fibrosis.

Collectively, these data revealed a promotive role of miR-125b in the development of liver fibrosis.

miR-125b Promotes HSC Activation and Contraction

Next, we used mouse primary HSCs to investigate the effect of miR-125b on the activation of HSCs. Primary HSCs were isolated from normal murine livers and then activated by culture for 7 days (Figure 2A). The activated HSCs displayed a significant increase in the levels of miR-125b (Figure 2B) and signature molecules of activated HSCs, such as *α-SMA*, *Col1a1*, *Col1a2*, *Timp1*, and *Fn1* (Figure 2C). Of note, inhibition of cellular miR-125b reduced the expression of *α-SMA*, *Col1a1*, *Col1a2*, and *Timp1* in the culture-activated HSCs (Figures 2D and 2E).

Transforming growth factor β (TGF-β) is a key pro-fibrogenic cytokine that promotes activation of HSCs.[1] Interestingly, TGF-β treatment promoted miR-125b expression (Figure 3A), whereas knockdown of TGF-β receptor type I (*Tgfbr1*), *Smad2*, or *Smad3* (Figure S6A) significantly reduced the TGF-β-induced miR-125b expression in JS1 cells (Figure 3B). As expected, inhibition of miR-125b (Figure S6B) abrogated the TGF-β-induced expression of *Col1a1*, *Col1a2* (Figure 3C), and *α-SMA* (Figure 3D). Even in the absence of TGF-β, the basal mRNA and protein levels of α-SMA were reduced in the anti-miR-125b-transfected JS1 cells (Figure 3E), but enhanced in the miR-125b-overexpressing cells (Figure 3F; Figure S6C). However, inhibition of miR-125b did not change the expression of *Col1a1* and *Col1a2* in JS1 cells without TGF-β stimulation (Figure S7). Taken together, these data indicate a promoting effect of miR-125b in the activation of HSCs.

It is well known that α-SMA promotes contraction of activated HSCs and consequently increases ECM stiffness.[4,16] Collagen gel contraction assay was therefore employed to evaluate the contractility of HSCs embedded in collagen matrix. The results showed that the size of gel-containing anti-NC (negative control RNA duplexes)-transfected

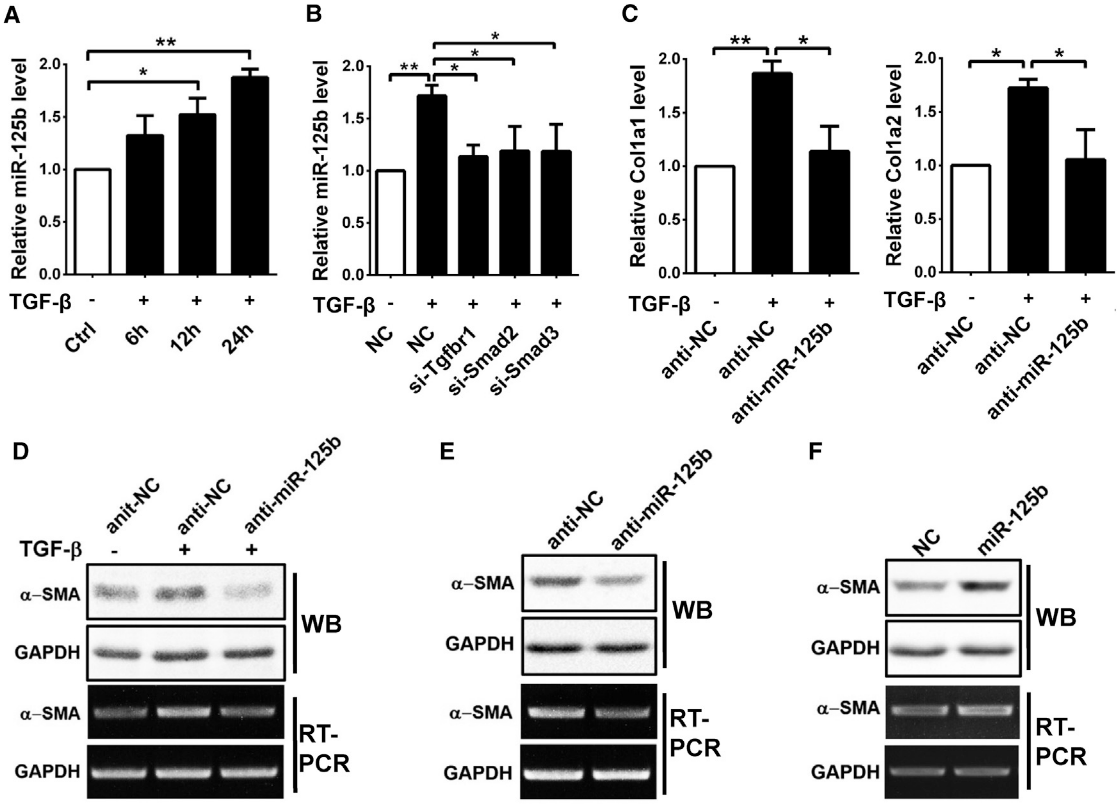

Figure 3. miR-125b Promotes Activation of JS1 Cells

(A) TGF-β treatment promoted miR-125b expression. JS1 cells without (–) or with (+) treatment of 5 ng/mL TGF-β for the indicated time periods were subjected to qRT-PCR analysis for miR-125b expression. (B) Silencing *Tgfbr1*, *Smad2*, or *Smad3* blocked the TGF-β-induced miR-125b expression. JS1 cells transfected with NC (negative control), si-Tgfbr1, si-Smad2, or si-Smad3 were incubated without (–) or with (+) TGF-β for 24 hr, followed by qRT-PCR analysis. (C and D) Knockdown of miR-125b blocked the TGF-β-induced *Col1a1*, *Col1a2*, and *α-SMA* expression. JS1 cells were transfected with anti-NC or anti-miR-125b for 48 hr, then incubated without (–) or with (+) 5 ng/mL TGF-β for 24 hr, followed by qRT-PCR (C), RT-PCR (D), or western blotting (D) analysis. (E) Knockdown of miR-125b decreased the basal level of *α-SMA*. (F) Overexpression of miR-125b increased the basal level of *α-SMA*. For (E) and (F), JS1 cells were transfected with the indicated RNA for 48 hr and then subjected to RT-PCR or western blotting analysis. Data are presented as mean ± SEM in (A)–(C). *p < 0.05; **p < 0.01.

JS1 cells became smaller after TGF-β treatment (Figure 4A), indicating that TGF-β promotes JS1 contraction. Interestingly, the TGF-β-promoted contraction was attenuated when miR-125b in JS1 cells was antagonized by anti-miR-125b (Figure 4A). Moreover, in the absence of TGF-β, anti-miR-125b-transfected JS1 cells displayed weaker contraction (Figure 4B), whereas miR-125b-overexpressing cells showed much stronger contractility (Figure 4C).

These data suggest that miR-125b may promote liver fibrosis by enhancing α-SMA expression and HSC contraction.

miR-125b Promotes α-SMA Expression and Enhances HSC Contraction by Directly Targeting Stard13

We then investigated the underlying mechanism responsible for miR-125b function. Among the miR-125b targets predicted by TargetScan (http://www.targetscan.org), we focused on Stard13 (Figure S8) because it is a RhoA-specific GTPase-activating protein (GAP) that converts the active GTP-bound RhoA into inactive GDP-bound RhoA.[17,18] It is known that the active RhoA promotes release of Mrtf-A protein from G-actin in cytoplasm; the resulting free Mrtf-A was then translocated into the nucleus, where it forms a complex with transcriptional factor Srf and activates the transcription of target genes,[19] including *α-SMA*. We therefore evaluated whether the miR-125b-stimulated α-SMA expression and HSC contraction were dependent on the Stard13-RhoA signaling. As shown, Stard13 protein level was significantly reduced in the culture-activated HSCs (day 7; Figure 5A), which was inversely correlated with upregulation of miR-125b (Figure 2B). In addition, silencing *Stard13* by small interference RNA duplexes (siRNAs) in JS1 cells significantly enhanced α-SMA expression (Figure 5B) and cell contraction (Figure 5C), which phenocopied the effects of miR-125b overexpression (Figures 3F and 4C). Moreover, silencing of *Stard13* attenuated the suppressive effect of anti-miR-125b on contraction of JS1 cells, suggesting that the fibrosis-promoting effect of miR-125b is carried out through Stard13 (Figure 5D). Subsequent dual-luciferase reporter analysis showed that co-expression of miR-125b significantly suppressed the activity of

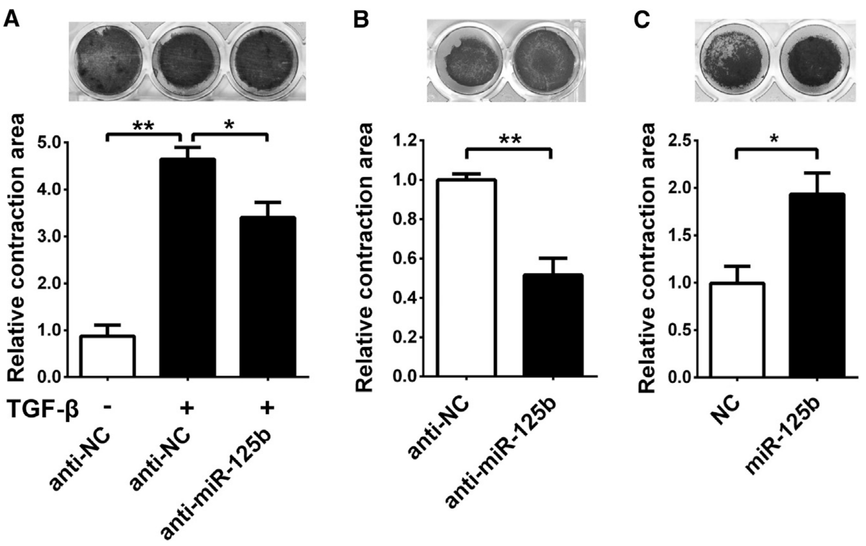

Figure 4. miR-125b Promotes the Contraction of JS1 Cells

(A) Inhibition of miR-125b attenuated the TGF-β-induced contraction of JS1 cells. The collagen lattice containing JS1 cells that were transfected with the indicated RNA was incubated without (−) or with (+) 5 ng/mL TGF-β for 24 hr, then released, followed by culture for another 24 hr before calculating contraction areas. (B) Antagonism of miR-125b inhibited the contraction of JS1 cells. (C) Ectopic expression of miR-125b promoted the contraction of JS1 cells. For (B) and (C), the collagen lattice containing JS1 cells that were transfected with miR-125b inhibitor (50 nM) or miR-125b mimic was incubated with 10% FBS-containing DMEM for 24 hr; the gels were then released, followed by culture for another 48 (B) or 24 (C) hr before measuring contraction areas. Data are presented as mean ± SEM in (A)–(C). *p < 0.05; **p < 0.01.

luciferase-containing wild-type, but not mutant, 3′ UTR of *Stard13* (Figure 5E; Figure S8). Furthermore, overexpression of miR-125b in JS1 cells reduced endogenous Stard13 protein, but not mRNA level, and repression of miR-125b increased the protein level of Stard13 (Figure 5F; Figure S9). These findings suggest that miR-125b may directly repress Stard13 expression in HSCs.

To further evaluate whether miR-125b affects the activation of RhoA, we determined the level of GTP-bound RhoA by GST-RBD (GST-Rho binding domain) pull-down assay. As shown, overexpression of miR-125b or knockdown of *Stard13* expression increased the GTP-RhoA level in JS1 cells (Figure 6A, left panel), whereas inhibition of miR-125b decreased the active RhoA level (Figure 6A, right panel). Moreover, knockdown of either *RhoA* (Figure 6B) or its downstream factors, *Mrtf-A* or *Srf* (Figure 6C; Figure S10), by RNAi significantly blocked the miR-125b-induced α-SMA expression. Consistently, silencing RhoA also blocked the miR-125b-induced JS1 cell contraction (Figure 6D).

Taken together, our findings suggest that expression of miR-125b is increased by TGF-β in activated HSCs, and the increased miR-125b binds to the 3′ UTR of *Stard13* and inhibits its expression, thereby enhancing the RhoA/Mrtf-A/Srf signaling and leading to the increased expression of α-SMA and type I collagen and augmented contraction of HSCs, consequently resulting in hepatic fibrosis (Figure 7).

DISCUSSION

In this study, we disclosed a miR-125b-Stard13-RhoA-α-SMA signaling pathway in HSCs and revealed a promotive role of miR-125b in HSC activation and liver fibrosis.

miR-125b is a ubiquitously expressed miRNA that plays important roles in cell proliferation, apoptosis, migration, and differentiation.[20] Previous studies have shown that miR-125b is also implicated in different liver diseases, including ALF,[8] non-alcoholic fatty liver disease,[9] cholestasis,[10] and HCC.[11,12] However, the function and its underlying mechanism of miR-125b in HSC activation and liver fibrosis remain unknown. Here, we disclosed an upregulation of miR-125b in HSCs during hepatic fibrogenesis *in vivo* (Figure 1C) and upon culture activation *in vitro* (Figure 2B). In addition, knockdown of miR-125b suppressed the activation of HSCs *in vitro* (Figures 2 and 3). Moreover, administration of miR-125b-sponge that antagonized endogenous miR-125b alleviated liver fibrosis in CCl_4-treated mice (Figures 1G and 1H). These data suggest a promotive role of miR-125b in HSC activation and liver fibrosis.

Activation of quiescent HSCs is a tightly orchestrated process that includes a number of functional changes, such as increasing proliferation, contractility, and synthesis of ECM components. To date, numerous miRNAs have been shown to regulate proliferation or apoptosis of HSCs, such as miR-150,[21] miR-122,[22] miR-15/16,[23] and miR-335,[24] while other miRNAs, like miR-133 and miR-29, are implicated in hepatic fibrosis by regulating production of ECM components.[25] However, no miRNA has been reported to regulate HSC contraction except miR-126b*.[26] It has been proposed that contractility of activated HSCs may lead to liver stiffness and hepatic sinusoidal vasoconstriction, and eventually cause liver complications, like portal hypertension.[27] Contractile force in activated HSCs is generated by the formation of stress fibers and phosphorylation of myosin light chain (myosin II).[28] RhoA/Rho-kinase signaling plays a critical role in HSC contraction.[29] Previous studies have shown that the expression of *α-SMA* is upregulated by RhoA/Mrtf-A/Srf signaling in smooth muscle cell,[30,31] and α-SMA can directly increase the contractility of fibroblasts by incorporating into stress fibers.[4,5] On the other hand, Rho-kinase increases the phosphorylation of myosin II by inhibiting myosin phosphatase or by promoting phosphorylation of myosin II, thereby enhancing the activity of myosin II and promoting cell contraction.[32] Stard13 is a RhoA-specific GAP that negatively regulates RhoA activation. Here, we showed that

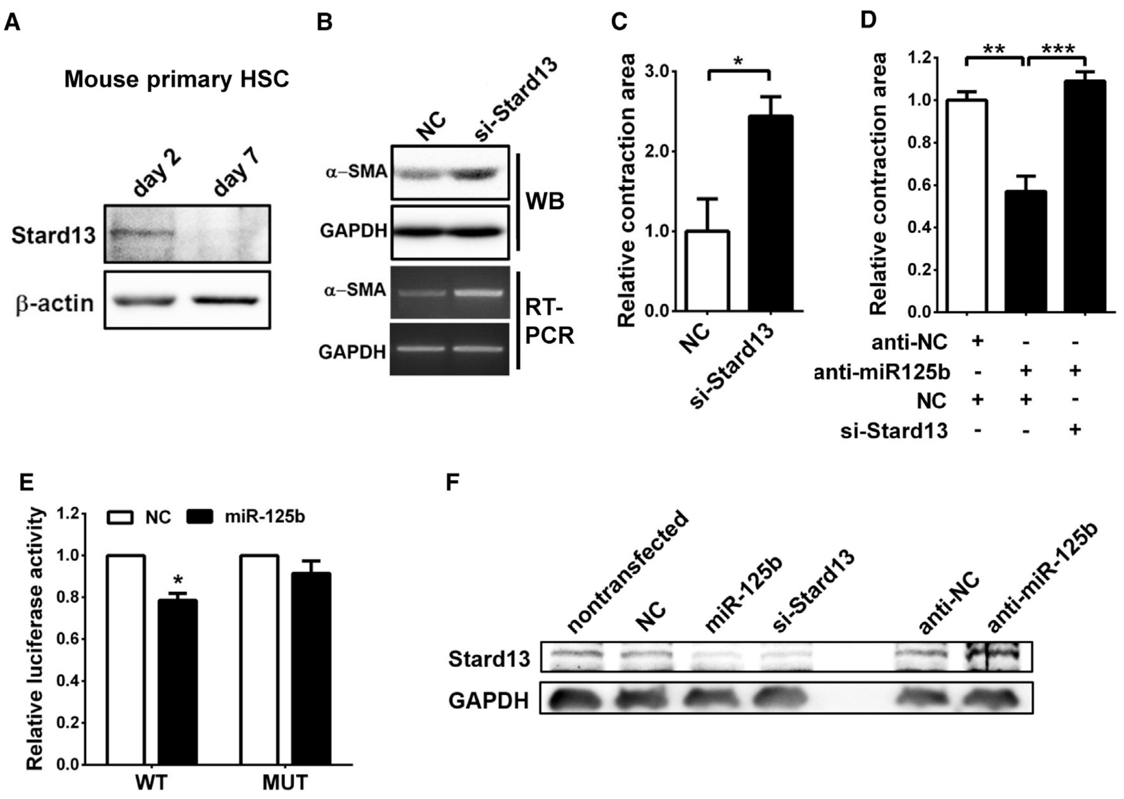

Figure 5. miR-125b Suppresses the Expression of Stard13 by Directly Binding to its 3′ UTR

(A) The protein level of Stard13 was decreased in the culture-activated HSCs. Mouse primary HSCs were cultured *in vitro* for 2 or 7 days before western blotting. (B) Knockdown of *Stard13* promoted *α-SMA* expression. JS1 cells were transfected with NC or si-Stard13 for 48 hr, followed by western blotting or RT-PCR analysis. (C) Inhibition of *Stard13* enhanced cell contraction. (D) Silencing of *Stard13* attenuated the suppressive effect of anti-miR-125b on JS1 contraction. For (C) and (D), the collagen lattice, which contained JS1 cells transfected with the indicated siRNA (50 nM) or anti-miR-125b (50 nM), was released and then incubated with 10% FBS-containing DMEM for 24 hr before measuring contraction area. (E) Overexpression of miR-125b suppressed the activity of luciferase reporter containing wild-type, but not mutant, 3′ UTR of *Stard13*. JS1 cells were cotransfected with NC or miR-125b duplexes and luciferase reporter plasmids that contained either wild-type (WT) or mutant (MUT) 3′ UTR of *Stard13* for 48 hr before luciferase assay. The Renilla luciferase activity of each sample was normalized to that of firefly luciferase. (F) Overexpression of miR-125b reduced cellular Stard13 level, and knockdown of endogenous miR-125b increased Stard13 expression. JS1 cells without transfection (lane 1) or transfected with the indicated RNA were incubated for 48 hr before western blotting analysis. Data are presented as mean ± SEM in (E)–(E). *p < 0.05; **p < 0.01; ***p < 0.001.

miR-125b directly suppressed Stard13 expression and consequently increased the level of active RhoA in HSCs (Figures 5F and 6A). Moreover, knockdown of *RhoA* or its downstream factors, *Mrtf-A* and *Srf*, significantly antagonized the miR-125b-induced α-SMA expression (Figures 6B and 6C), and inhibition of *RhoA* expression suppressed the miR-125b-promoted HSC contraction (Figure 6D). Furthermore, silencing *Stard13* attenuated the suppressive effect of anti-miR-125b on HSC contraction (Figure 5D). Therefore, our findings suggest that miR-125b promotes α-SMA expression and cell contraction by targeting the Stard13/RhoA signaling pathway. As shown, inhibition of miR-125b reduced the level of α-SMA in culture-activated primary HSCs (Figure 2D) and in JS1 cells with (Figure 3D) and without TGF-β treatment (Figure 3E). On the other hand, knockdown of miR-125b resulted in a decrease of *Col1a1* and *Col1a2* in culture-activated primary HSCs (Figure 2D) and in TGF-β-treated JS1 cells (Figure 3C), but not in JS1 cells, without TGF-β treatment (Figure S7). Therefore, we focused on exploring whether miR-125 promoted fibrosis by regulating α-SMA. However, we cannot exclude the possibility that miR-125b may promote liver fibrosis by enhancing Col1a1 and Col1a2 expression.

TGF-β is the most prominent fibrogenic cytokine in HSC activation. It also regulates the expression of several fibrosis-related miRNAs, such as miR-29,[13] miR-133a,[25] and miR-21.[33] Here, we revealed that TGF-β treatment significantly increased the level of miR-125b in HSCs. Moreover, knockdown of *Tgfbr1*, *Smad2*, or *Smad3* blocked the TGF-β-induced miR-125b expression. Notably, inhibition of miR-125b significantly blocked the TGF-β-induced α-SMA expression and HSC contraction. These findings suggest that TGF-β may activate HSCs by increasing miR-125b level during liver fibrogenesis.

At the early phase of CCl_4 treatment, a significant number of fibroblasts undergo proliferation.[34] It is reported that efficient lentiviral

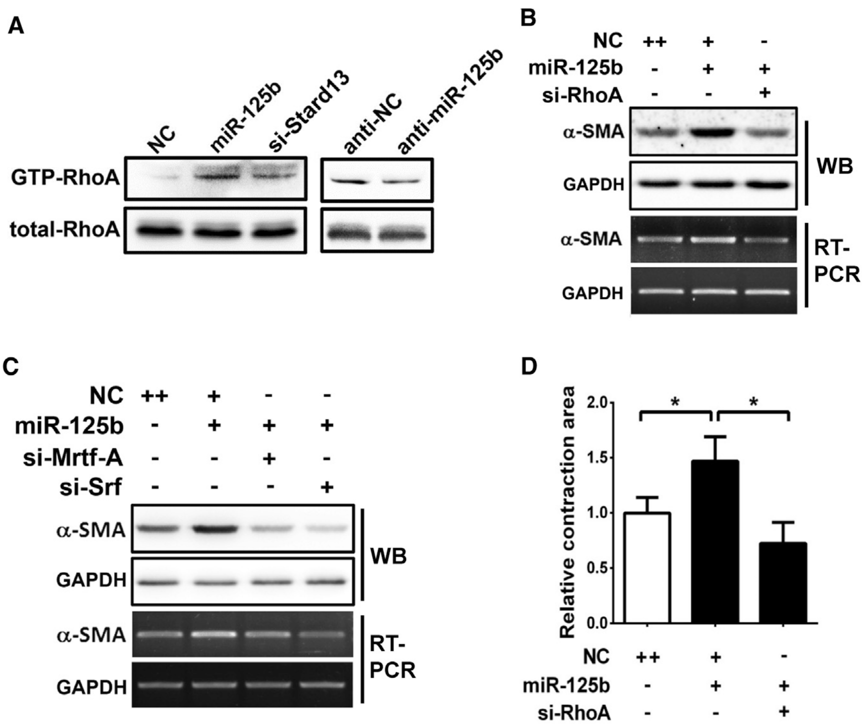

Figure 6. miR-125b Promoted α-SMA Expression and Cell Contraction by Enhancing RhoA Activity

(A) miR-125b enhanced RhoA activation. Lysates from JS1 cells that were transfected with the indicated RNA were incubated with the immobilized GST-RBD. The bead-bound proteins were dissolved in 2× Laemmli sample buffer and analyzed by immunoblotting for GTP-bound RhoA. Equal aliquots were used to detect total RhoA in each lysate. (B and C) Knockdown of *RhoA*, *Mrtf-A*, or *Srf* blocked the miR-125b-induced *α-SMA* expression. JS1 cells were cotransfected with miR-125b and the siRNA targeting *RhoA* (B), *Mrtf-A* or *Srf* (C) for 48 hr, followed by RT-PCR or western blotting analysis. (D) Silencing of *RhoA* blocked the miR-125b-induced contraction of JS1 cells. Collagen gel containing JS1 cells that were transfected with the indicated RNA was released and then incubated with 10% FBS-containing DMEM for 24 hr before analysis for contraction area. Data are presented as mean ± SEM in (D). *p < 0.05.

transduction in the livers requires cell proliferation *in vivo*,[35] although it has been shown that lentiviruses can also deliver genes into non-dividing cells.[36] This notion is supported by our finding that a number of activated HSCs were successfully infected with lentiviral miR-125-sponge in the CCl_4-treated fibrosis model (Figures S4A and S4B). In addition, infection of miR-125b-sponge-lentiviruses dramatically reduced the miR-125b level in mouse HSCs *in vitro* (Figure S3) and in CCl_4-treated livers (Figure S5) *in vivo*. These findings suggest that miR-125b-sponge-lentiviruses may alleviate liver fibrosis by inactivating HSCs.

In summary, our findings uncover a miR-125b-Stard13-RhoA-α-SMA signaling cascade in HSCs and highlight its importance in hepatic fibrosis.

MATERIALS AND METHODS

Human Tissues

Human fibrotic liver tissues were collected from five HCC patients who underwent curative resection at the Cancer Center of Sun Yat-sen University and four hepatic failure patients with liver cirrhosis who underwent liver transplantation at the Third Affiliated Hospital of Sun Yat-sen University. The normal liver tissues were collected from nine patients undergoing resection of hepatic hemangiomas at the Cancer Center of Sun Yat-sen University. Informed consent was obtained from each patient, and the study was approved by the Institute Research Ethics Committee. Characteristics of liver fibrosis and hepatic hemangioma patients are shown in Table S1.

Mouse Liver Fibrosis Models

Male C57BL/6 mice at 6 weeks of age were purchased from Guangdong Medical Laboratory Animal Center. Two mouse models for liver fibrosis were used in this study. In the model of chemical-induced liver fibrosis, CCl_4 was dissolved in corn oil (1:5, v/v) and intraperitoneally injected into mouse at a dose of 0.5 mL/kg body weight, twice a week for 4 or 6 weeks. Corn oil-injected mice served as controls. In the BDL model, the common bile duct of mouse was ligated for 21 days. Sham-operated mice served as controls. For *in vivo* viral infection assay, mice were intravenously injected with lentiviruses (~1.6–1.7 × 10^{10} vg resuspended in 100 μL of 1× PBS). All animal experiment procedures were performed in accordance with the Guide for the Care and Use of Laboratory Animals (NIH publications No. 80-23, revised 1996) and according to the institutional ethical guidelines for animal experiments.

Cell Line and Primary Cells

The immortalized mouse HSC line, JS1[37,38] (kindly gifted by Dr. Jinsheng Guo from Zhongshan Hospital at Fudan University, Shanghai), was maintained in DMEM complemented with 10% fetal bovine serum.

Mouse primary HSCs were isolated from male C57BL/6 mice by collagenase perfusion and density gradient centrifugation as previously described.[39] In brief, liver was perfused sequentially with ethylene glycol-bis (2-aminoethylether)-*N,N,N',N'*-tetraacetic acid (EGTA)-containing D-Hank's buffer for 5 min, then with 0.25 mg/mL collagenase I-containing Hank's buffer for 20 min. The perfused liver was transferred to a beaker, minced, and digested with 0.02 mg/mL DNase I-containing Hank's buffer for 10 min at 37°C with slowly stirring. The resulting cell homogenates were filtered through a 100-μm cell strainer followed by washing twice

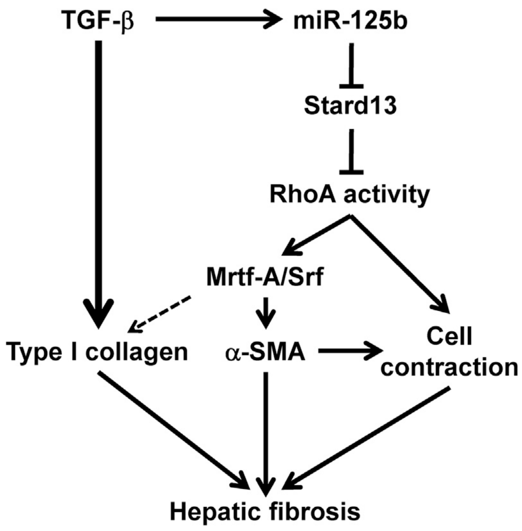

Figure 7. Schematic Representation of the Function of miR-125b in HSC Activation and Liver Fibrosis

with ice-cold Hank's buffer. Cell pellets were re-suspended in 15% OptiPrep (Alere Technologies, Norway) and topped with 11.5% OptiPrep and then with 1× PBS. After centrifuging at 1,400 × *g*, 4°C for 17 min, the viable HSCs between layers of 11.5% OptiPrep and 1× PBS were collected and then washed once with ice-cold 1× PBS. The purity of the isolated HSCs was assessed by retinoid autofluorescence. HSCs with purity over 90% were used.

To isolate primary hepatocytes, we washed cell homogenates with ice-cold Hank's, then re-suspended in 50% Percoll (diluted by Hank's buffer; GE Healthcare, Chicago, IL, USA) and centrifuged at 50 × *g*, 4°C for 15 min. The isolated hepatocytes were washed with ice-cold Hank's buffer. Both primary HSCs and hepatocytes were cultured in DMEM supplemented with 10% fetal bovine serum and 1% penicillin-streptomycin.

RNA Oligoribonucleotides and Vectors

miR-125b mimic, siRNAs, negative control RNA duplexes (NC), and the sequence-specific RNA oligoribonucleotide of miR-125b inhibitor (anti-miR-125b) and its control (anti-NC) with 2′-O-methyl modification were purchased from Genepharma (Shanghai, China). Sequences of all RNA oligoes used in this study are listed in Table S2.

For vectors, a psiCHECK-2 (cat. C8021; Promega, Madison, WI, USA) vector was used to construct luciferase reporter plasmids, including psiCHECK2-Stard13-3′UTR-WT and psiCHECK2-Stard13-3′ UTR-MUT, which contained a wild-type and mutant 3′ UTR segment of mouse Stard13 gene, respectively.

A lentiviral vector with miR-125b-sponge, which contained six tandem repeats of miR-125b binding sequence following the stop codon of *mCherry* gene, was used. The fused mCherry-miR-125b-sponge sequence was PCR amplified from ED214 plasmid[40] (kindly gifted by Dr. Dieter Edbauer from the Picower Institute for Learning and Memory, USA) and inserted into the multiple cloning sites of pCDH-CMV-MCS-EF1-copGFP vector (cat. CD511B-1; System Biosciences, Palo Alto, CA, USA). A control vector (Ctrl) that only contained mCherry expression cassette without miR-125b binding sequence was also generated.

Sequences of all primers used for plasmids construction are listed in Table S2.

Immunohistochemical, Immunofluorescent, and Sirius Red Staining

Mouse liver sections (4 μm thick) from paraffin-embedded samples were applied to immunohistochemical staining using monoclonal antibody (mAb) against α-SMA (cat. ZM-0003; ZSGB-BIO, Beijing, China) and polyclonal antibody (pAb) against copGFP (cat. AB513; Evrogen, Russia) to identify activated HSCs and lentivirus-infected cells, respectively. Fibrillar collagen was detected by Sirius red staining. To evaluate the expression of α-SMA and deposition of collagen, we acquired 10 areas at a magnification of ×100 under a light microscope for each section. The positive staining area was digitized by Image Pro Plus software (Version 6.0; Media Cybernetics, Bethesda, MD, USA) using color cube-based selection criteria.

To perform immunofluorescent staining, we stained frozen sections (8 μm thick) from lentivirus-infected livers, which displayed copGFP fluorescent signals (green), with mAb against α-SMA (cat. ab124964; Abcam, Cambridge, MA, USA) and with DAPI for nuclei. An Alexa Fluor 647-conjugated goat anti-Rabbit IgG pAb (cat. A-21245; Invitrogen, Waltham, MA, USA) was used as a secondary antibody. Images were captured by laser scanning confocal microscope.

Analysis of Gene Expression

Semiquantitative RT-PCR was used to analyze α-SMA expression, and GAPDH was used as an internal control. Real-time qRT-PCR was used to analyze the expression of profibrogenic genes. Sequences of all primers used for RT-PCR or qRT-PCR are listed in Table S2. miR-125b expression was analyzed using TaqMan MicroRNA Assays (Applied Biosystems, Foster City, CA, USA).

Antibodies for western blotting were Rabbit pAbs for α-SMA (cat. ab5694; Abcam, Cambridge, UK) and Stard13 (cat. Sc-135273; Santa Cruz Biotechnology, Santa Cruz, CA, USA), and mouse mAbs for β-actin (cat. BM0627; Boster, Wuhan, China) and GAPDH (cat. TA802519; OriGene, Rockville, MD, USA).

Cell Transfection

Reverse transfections of RNA oligoribonucleotides were performed with Lipofectamine RNAiMAX (cat. 13778150; Invitrogen, Waltham, MA, USA). siRNAs targeting different regions of each gene were pooled together with an equal molar amount and transfected into cells at a final concentration of 50 nM. Unless otherwise indicated, a final concentration of 200 nM for miRNA inhibitors was used.

Transfection of plasmid DNA was performed with Lipofectamine 2000 (cat. 11668019; Invitrogen, Waltham, MA, USA).

Packaging of the lentiviruses for miR-125b-sponge expression was performed in HEK293T cells by calcium phosphate transfection.

Cell Contraction Assay

JS1 (4×10^4 cells) were mixed with rat-tail collagen (final concentration 1 mg/mL), incubated for 1 hr at 37°C in a 48-well plate to allow gelation, and then maintained in 10% FBS-containing DMEM for 24 hr. To initiate contraction, we detached the gel lattice from the cell culture plate and incubated for another 24 or 48 hr before staining with crystal violet. The radius (r) of collagen gel was measured, and the area (S) was calculated using the formula: $S = \pi \times r^2$. The contraction area was defined as the reduced collagen gel surface area after collagen lattice release. The mean value of contraction areas from multiple independent experiments in the control group was set to 1. Relative contraction area is presented.

RhoA Activation Assay

Recombinant GST-RBD fusion proteins were purified from *Escherichia coli* that had been transformed with pGEX-2T-GST-RBD plasmid and immobilized on glutathione-Sepharose 4B beads (cat. C600031; Sangon Biotech, Shanghai, China), as described previously.[41] For the GST pull-down assay, JS1 cells were washed once with ice-cold 1× PBS and lysed on ice using eukaryotic cell lysis buffer (25 mM HEPES [pH 7.5], 150 mM NaCl, 1% NP-40, 10 mM $MgCl_2$, 1 mM EDTA, 2% glycerol) supplemented with protease inhibitors. The cell lysates were then incubated with immobilized GST-RBD for 2 hr at 4°C. The bead-immunocomplexes were washed three times with eukaryotic cell lysis buffer, and the bead-bound proteins were eluted with SDS buffer and analyzed by immunoblotting.

Hydroxyproline Assay

Collagen deposition of the liver was quantified by determining hydroxyproline content using the Hydroxyproline Colorimetric Assay Kit (cat. K555-100; BioVision, Milpitas, CA, USA). In brief, frozen liver tissues were hydrolyzed with 6 N HCl at 120°C for 3 hr. After brief cooling on ice and centrifugation at 10,000 × *g* for 3 min, 10 μL of the supernatant was transferred to a 96-well plate and incubated at 80°C until complete desiccation. Then 100 μL chloramine T reagent was added to each sample and incubated at room temperature for 5 min, followed by adding 100 μL of 4-dimethylaminobenzaldehyde (DMAB) reagent and incubation at 60°C for 90 min. After cooling to room temperature, absorbance at 560 nm was measured in a microplate reader (Infinite M200 Plate Reader; Tecan, Männedorf, Switzerland). Hydroxyproline content was expressed as micrograms per gram (μg/g) of liver tissue.

Statistical Analysis

The data are expressed as the mean ± SEM from at least three independent experiments. The differences between the groups were analyzed using Student's t test when two groups were compared or by one-way ANOVA when more than two groups were compared. Analyses were performed using the GraphPad Prism program (version 6.0; GraphPad Software, San Diego, CA, USA). All statistical tests were two-sided, and $p < 0.05$ was considered to be statistically significant.

SUPPLEMENTAL INFORMATION

Supplemental Information includes ten figures and two tables and can be found with this article online at https://doi.org/10.1016/j.omtn.2018.04.016.

AUTHOR CONTRIBUTIONS

Conception and Design: K.Y., J.Y., S.-M.Z.; Acquisition of Data: K.Y., S.-Y.L.; Analysis and Interpretation of Data: K.Y., J.Y., S.-M.Z.; Writing – Review and/or Revision of the Manuscript: K.Y., J.Y., S.-M.Z.; Administrative, Technical, or Material Support: J.G., J.-H.F., C.Z., M.Z., Y.Y.; Study Supervision: S.-M.Z.

CONFLICTS OF INTEREST

No conflicts of interest were declared.

ACKNOWLEDGMENTS

We thank Dr. Jinsheng Guo for the gift of immortalized mouse hepatic stellate cell line (JS1) and Dr. Dieter Edbauer for the gift of ED214 plasmid. This work was supported by grants from the National Key R&D Program of China (2017YFA0504400), the National Natural Science Foundation of China (91440205, 81572400), the Natural Science Foundation of Guangdong Province (2017A030313107), and the Fundamental Research Funds for the Central Universities (171gjc32).

REFERENCES

1. Bataller, R., and Brenner, D.A. (2005). Liver fibrosis. J. Clin. Invest. *115*, 209–218.
2. Hernandez-Gea, V., and Friedman, S.L. (2011). Pathogenesis of liver fibrosis. Annu. Rev. Pathol. *6*, 425–456.
3. Zhang, D.Y., and Friedman, S.L. (2012). Fibrosis-dependent mechanisms of hepatocarcinogenesis. Hepatology *56*, 769–775.
4. Hinz, B., Celetta, G., Tomasek, J.J., Gabbiani, G., and Chaponnier, C. (2001). Alpha-smooth muscle actin expression upregulates fibroblast contractile activity. Mol. Biol. Cell *12*, 2730–2741.
5. Chaponnier, C., Goethals, M., Janmey, P.A., Gabbiani, F., Gabbiani, G., and Vandekerckhove, J. (1995). The specific NH2-terminal sequence Ac-EEED of alpha-smooth muscle actin plays a role in polymerization in vitro and in vivo. J. Cell Biol. *130*, 887–895.
6. Soon, R.K., Jr., and Yee, H.F., Jr. (2008). Stellate cell contraction: role, regulation, and potential therapeutic target. Clin. Liver Dis. *12*, 791–803.
7. Tsochatzis, E.A., Bosch, J., and Burroughs, A.K. (2014). Liver cirrhosis. Lancet *383*, 1749–1761.
8. Yang, D., Yuan, Q., Balakrishnan, A., Bantel, H., Klusmann, J.H., Manns, M.P., Ott, M., Cantz, T., and Sharma, A.D. (2016). MicroRNA-125b-5p mimic inhibits acute liver failure. Nat. Commun. *7*, 11916.
9. Zhang, Z.C., Liu, Y., Xiao, L.L., Li, S.F., Jiang, J.H., Zhao, Y., Qian, S.W., Tang, Q.Q., and Li, X. (2015). Upregulation of miR-125b by estrogen protects against non-alcoholic fatty liver in female mice. J. Hepatol. *63*, 1466–1475.
10. Meng, F., Onori, P., Hargrove, L., Han, Y., Kennedy, L., Graf, A., Hodges, K., Ueno, Y., Francis, T., Gaudio, E., and Francis, H.L. (2014). Regulation of the histamine/VEGF axis by miR-125b during cholestatic liver injury in mice. Am. J. Pathol. *184*, 662–673.

11. Liang, L., Wong, C.M., Ying, Q., Fan, D.N., Huang, S., Ding, J., Yao, J., Yan, M., Li, J., Yao, M., et al. (2010). MicroRNA-125b suppressesed human liver cancer cell proliferation and metastasis by directly targeting oncogene LIN28B2. Hepatology *52*, 1731–1740.

12. Gong, J., Zhang, J.P., Li, B., Zeng, C., You, K., Chen, M.X., Yuan, Y., and Zhuang, S.M. (2013). MicroRNA-125b promotes apoptosis by regulating the expression of Mcl-1, Bcl-w and IL-6R. Oncogene *32*, 3071–3079.

13. Roderburg, C., Urban, G.W., Bettermann, K., Vucur, M., Zimmermann, H., Schmidt, S., Janssen, J., Koppe, C., Knolle, P., Castoldi, M., et al. (2011). Micro-RNA profiling reveals a role for miR-29 in human and murine liver fibrosis. Hepatology *53*, 209–218.

14. Giray, B.G., Emekdas, G., Tezcan, S., Ulger, M., Serin, M.S., Sezgin, O., Altintas, E., and Tiftik, E.N. (2014). Profiles of serum microRNAs; miR-125b-5p and miR223-3p serve as novel biomarkers for HBV-positive hepatocellular carcinoma. Mol. Biol. Rep. *41*, 4513–4519.

15. Ebert, M.S., Neilson, J.R., and Sharp, P.A. (2007). MicroRNA sponges: competitive inhibitors of small RNAs in mammalian cells. Nat. Methods *4*, 721–726.

16. Wang, J., Fan, J., Laschinger, C., Arora, P.D., Kapus, A., Seth, A., and McCulloch, C.A. (2005). Smooth muscle actin determines mechanical force-induced p38 activation. J. Biol. Chem. *280*, 7273–7284.

17. Ching, Y.P., Wong, C.M., Chan, S.F., Leung, T.H., Ng, D.C., Jin, D.Y., and Ng, I.O. (2003). Deleted in liver cancer (DLC) 2 encodes a RhoGAP protein with growth suppressor function and is underexpressed in hepatocellular carcinoma. J. Biol. Chem. *278*, 10824–10830.

18. Leung, T.H., Ching, Y.P., Yam, J.W., Wong, C.M., Yau, T.O., Jin, D.Y., and Ng, I.O. (2005). Deleted in liver cancer 2 (DLC2) suppresses cell transformation by means of inhibition of RhoA activity. Proc. Natl. Acad. Sci. USA *102*, 15207–15212.

19. Zhao, X., Ding, E.Y., Yu, O.M., Xiang, S.Y., Tan-Sah, V.P., Yung, B.S., Hedgpeth, J., Neubig, R.R., Lau, L.F., Brown, J.H., and Miyamoto, S. (2014). Induction of the matricellular protein CCN1 through RhoA and MRTF-A contributes to ischemic cardioprotection. J. Mol. Cell. Cardiol. *75*, 152–161.

20. Sun, Y.M., Lin, K.Y., and Chen, Y.Q. (2013). Diverse functions of miR-125 family in different cell contexts. J Hematol Oncol. *6*, 6.

21. Zheng, J., Lin, Z., Dong, P., Lu, Z., Gao, S., Chen, X., Wu, C., and Yu, F. (2013). Activation of hepatic stellate cells is suppressed by microRNA-150. Int. J. Mol. Med. *32*, 17–24.

22. Li, J., Ghazwani, M., Zhang, Y., Lu, J., Li, J., Fan, J., Gandhi, C.R., and Li, S. (2013). miR-122 regulates collagen production via targeting hepatic stellate cells and suppressing P4HA1 expression. J. Hepatol. *58*, 522–528.

23. Guo, C.J., Pan, Q., Li, D.G., Sun, H., and Liu, B.W. (2009). miR-15b and miR-16 are implicated in activation of the rat hepatic stellate cell: an essential role for apoptosis. J. Hepatol. *50*, 766–778.

24. Chen, C., Wu, C.Q., Zhang, Z.Q., Yao, D.K., and Zhu, L. (2011). Loss of expression of miR-335 is implicated in hepatic stellate cell migration and activation. Exp. Cell Res. *317*, 1714–1725.

25. Roderburg, C., Luedde, M., Vargas Cardenas, D., Vucur, M., Mollnow, T., Zimmermann, H.W., Koch, A., Hellerbrand, C., Weiskirchen, R., Frey, N., et al. (2013). miR-133a mediates TGF-β-dependent derepression of collagen synthesis in hepatic stellate cells during liver fibrosis. J. Hepatol. *58*, 736–742.

26. Guo, C.-J., Pan, Q., Xiong, H., Qiao, Y.-Q., Bian, Z.-L., Zhong, W., Sheng, L., Li, H., Shen, L., Hua, J., et al. (2013). Dynamic expression of miR-126* and its effects on proliferation and contraction of hepatic stellate cells. FEBS Lett. *587*, 3792–3801.

27. Friedman, S.L. (2008). Hepatic stellate cells: protean, multifunctional, and enigmatic cells of the liver. Physiol. Rev. *88*, 125–172.

28. Narumiya, S., Tanji, M., and Ishizaki, T. (2009). Rho signaling, ROCK and mDia1, in transformation, metastasis and invasion. Cancer Metastasis Rev. *28*, 65–76.

29. Laleman, W., Van Landeghem, L., Severi, T., Vander Elst, I., Zeegers, M., Bisschops, R., Van Pelt, J., Roskams, T., Cassiman, D., Fevery, J., and Nevens, F. (2007). Both Ca2+-dependent and -independent pathways are involved in rat hepatic stellate cell contraction and intrahepatic hyperresponsiveness to methoxamine. Am. J. Physiol. Gastrointest. Liver Physiol. *292*, G556–G564.

30. Du, K.L., Ip, H.S., Li, J., Chen, M., Dandre, F., Yu, W., Lu, M.M., Owens, G.K., and Parmacek, M.S. (2003). Myocardin is a critical serum response factor cofactor in the transcriptional program regulating smooth muscle cell differentiation. Mol. Cell. Biol. *23*, 2425–2437.

31. Mack, C.P., Somlyo, A.V., Hautmann, M., Somlyo, A.P., and Owens, G.K. (2001). Smooth muscle differentiation marker gene expression is regulated by RhoA-mediated actin polymerization. J. Biol. Chem. *276*, 341–347.

32. Reynaert, H., Urbain, D., and Geerts, A. (2008). Regulation of sinusoidal perfusion in portal hypertension. Anat. Rec. (Hoboken) *291*, 693–698.

33. Liu, X., Hong, Q., Wang, Z., Yu, Y., Zou, X., and Xu, L. (2016). Transforming growth factor-β-sphingosine kinase 1/S1P signaling upregulates microRNA-21 to promote fibrosis in renal tubular epithelial cells. Exp. Biol. Med. (Maywood) *241*, 265–272.

34. Desmoulière, A., Xu, G., Costa, A.M.A., Yousef, I.M., Gabbiani, G., and Tuchweber, B. (1999). Effect of pentoxifylline on early proliferation and phenotypic modulation of fibrogenic cells in two rat models of liver fibrosis and on cultured hepatic stellate cells. J. Hepatol. *30*, 621–631.

35. Park, F., Ohashi, K., Chiu, W., Naldini, L., and Kay, M.A. (2000). Efficient lentiviral transduction of liver requires cell cycling in vivo. Nat. Genet. *24*, 49–52.

36. Naldini, L. (1998). Lentiviruses as gene transfer agents for delivery to non-dividing cells. Curr. Opin. Biotechnol. *9*, 457–463.

37. Guo, J., Loke, J., Zheng, F., Hong, F., Yea, S., Fukata, M., Tarocchi, M., Abar, O.T., Huang, H., Sninsky, J.J., and Friedman, S.L. (2009). Functional linkage of cirrhosis-predictive single nucleotide polymorphisms of Toll-like receptor 4 to hepatic stellate cell responses. Hepatology *49*, 960–968.

38. Zhang, Z., Lin, C., Peng, L., Ouyang, Y., Cao, Y., Wang, J., Friedman, S.L., and Guo, J. (2012). High mobility group box 1 activates Toll like receptor 4 signaling in hepatic stellate cells. Life Sci. *91*, 207–212.

39. Zeng, C., Wang, Y.L., Xie, C., Sang, Y., Li, T.J., Zhang, M., Wang, R., Zhang, Q., Zheng, L., and Zhuang, S.M. (2015). Identification of a novel TGF-β-miR-122-fibronectin 1/serum response factor signaling cascade and its implication in hepatic fibrogenesis. Oncotarget *6*, 12224–12233.

40. Edbauer, D., Neilson, J.R., Foster, K.A., Wang, C.F., Seeburg, D.P., Batterton, M.N., Tada, T., Dolan, B.M., Sharp, P.A., and Sheng, M. (2010). Regulation of synaptic structure and function by FMRP-associated microRNAs miR-125b and miR-132. Neuron *65*, 373–384.

41. Guilluy, C., Dubash, A.D., and García-Mata, R. (2011). Analysis of RhoA and Rho GEF activity in whole cells and the cell nucleus. Nat. Protoc. *6*, 2050–2060.

OMTN, Volume *12*

Supplemental Information

MicroRNA-125b Promotes Hepatic Stellate Cell Activation and Liver Fibrosis by Activating RhoA Signaling

Kai You, Song-Yang Li, Jiao Gong, Jian-Hong Fang, Chong Zhang, Min Zhang, Yunfei Yuan, Jine Yang, and Shi-Mei Zhuang

Supplemental Data:

Supplemental Figures

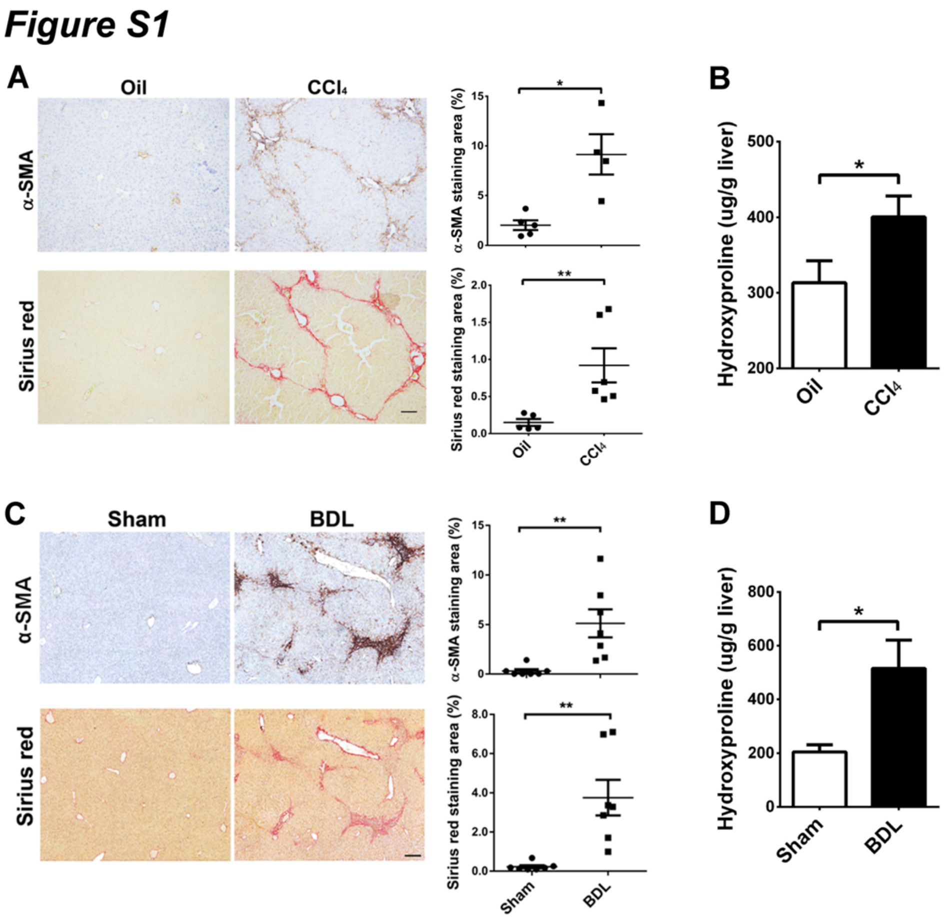

Figure S1. Induction of liver fibrosis in mice. (A-B) CCl_4 induced liver fibrosis in mice. Male C57BL/6 mice at 6 weeks of age were treated with CCl_4 (0.5 μL/g body weight) for 6 weeks. (C-D) BDL induced liver fibrosis in mice. Male C57BL/6 mice at 6 weeks of age were treated with bile duct ligation for 3 weeks. For (A) and (C), liver fibrosis was assessed by α-SMA and Sirius red staining (magnification, ×100). Percentage of positive staining area relative to total area was quantified by image pro plus version 6.0 software and shown in the *right*. For (B) and (D), hydroxyproline content of the livers was determined by Hydroxyproline Colorimetric Assay kit. Scale bar, 100 μm. Data are presented as mean± SEM in (A)-(D). *, $P < 0.05$; **, $P < 0.01$.

Figure S2

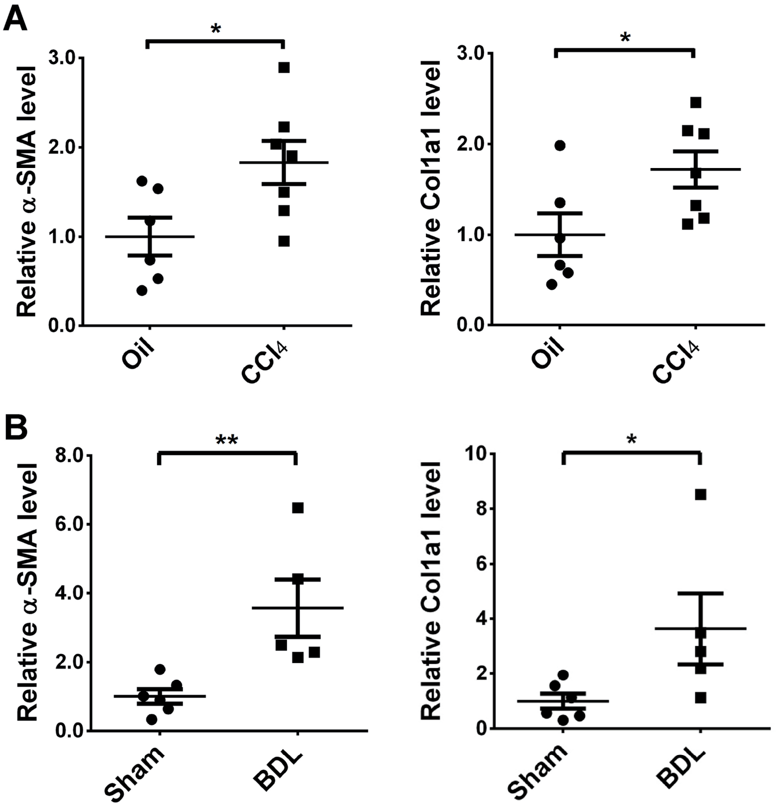

Figure S2. Expression of *α-SMA* and *Col1a1* was increased in the HSCs of fibrotic livers. HSCs were isolated from the fibrotic livers of mice treated with CCl_4 (A) or BDL (B) or from the normal livers of control mice, and then cultured for 24 hours before qRT-PCR analysis. The mean expression level of each gene in control group was set as relative level 1. Data are presented as mean ± SEM in (A)-(B). *, $P < 0.05$; **, $P < 0.01$.

Figure S3

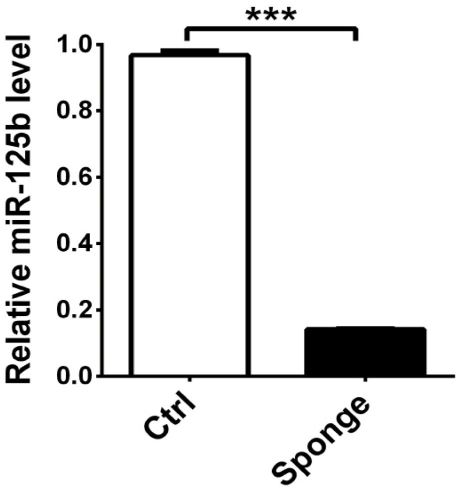

Figure S3. The level of miR-125b was decreased in the JS1 cells infected with miR-125b sponge-lentivirus. JS1 cells were infected by control (Ctrl) or miR-125b-sponge-lentivirus (Sponge) to establish stable infected cell lines. In passage 4, the expression level of miR-125b in both Ctrl and Sponge groups was analyzed by qRT-PCR. Data are presented as mean± SEM. *** $P < 0.001$.

Figure S4

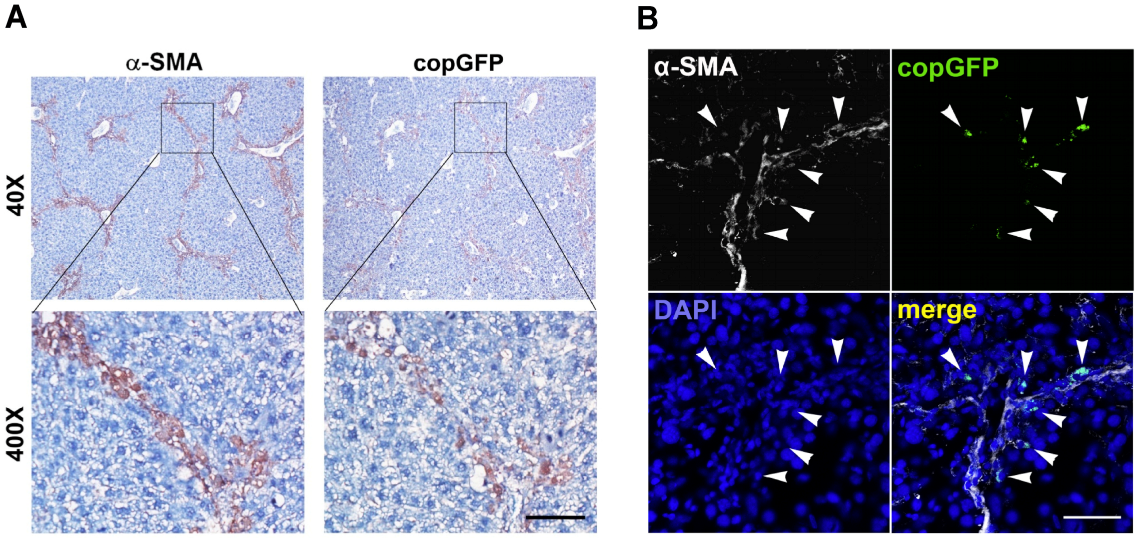

Figure S4. Activated HSCs were infected by lentiviruses *in vivo*. (A) Immunohistochemistry analysis was performed to determine the distribution of activated HSCs and the lentivirus-infected cells. Serial sections from the liver of CCl_4-treated mice, which were intravenously injected with lentiviruses, were stained with α-SMA and copGFP antibody, respectively. (B) Immunofluorescent staining was performed to determine the distribution of activated HSCs and the lentivirus-infected cells. Frozen liver sections were stained with α-SMA (white) and DAPI (blue). Lentivirus-infected cells showed green due to expression of copGFP. Arrowheads indicate the infection of lentiviruses in activated HSCs (co-localization of α-SMA and copGFP). Scale bar, 50 μm.

Figure S5

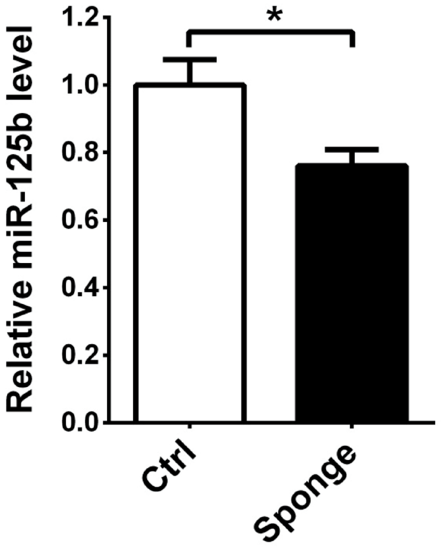

Figure S5. Administration of miR-125b-sponge reduced endogenous miR-125b level in mouse liver. miR-125b-sponge (Sponge) or its control (Ctrl) lentiviruses were injected through tail vein on the 5th and 7th day after the first administration of CCl_4. Mouse livers were collected and subjected to qRT-PCR analysis at 3 weeks after the last injection of lentiviruses. Data are presented as mean ± SEM. *, $P < 0.05$.

Figure S6

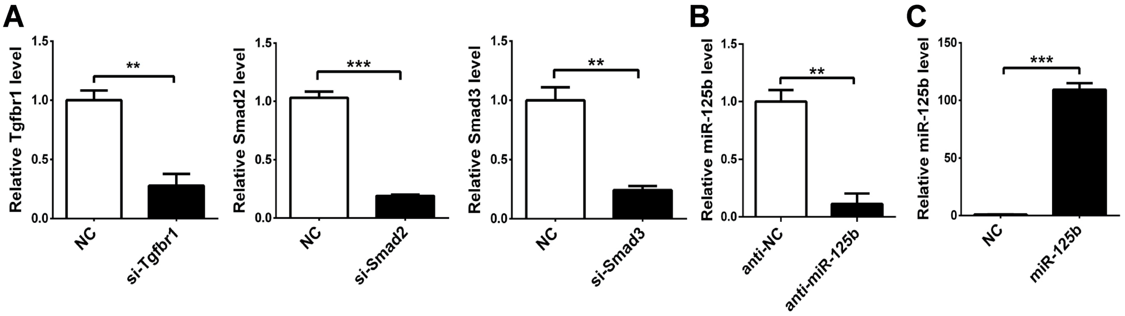

Figure S6. The transfection efficiency of RNA oligoribonucleotides. JS1 cells were transfected with the indicated RNA for 48 hours before qRT-PCR analysis. Data are presented as mean ± SEM in (A)-(C). **, $P < 0.05$; ***, $P < 0.001$.

Figure S7

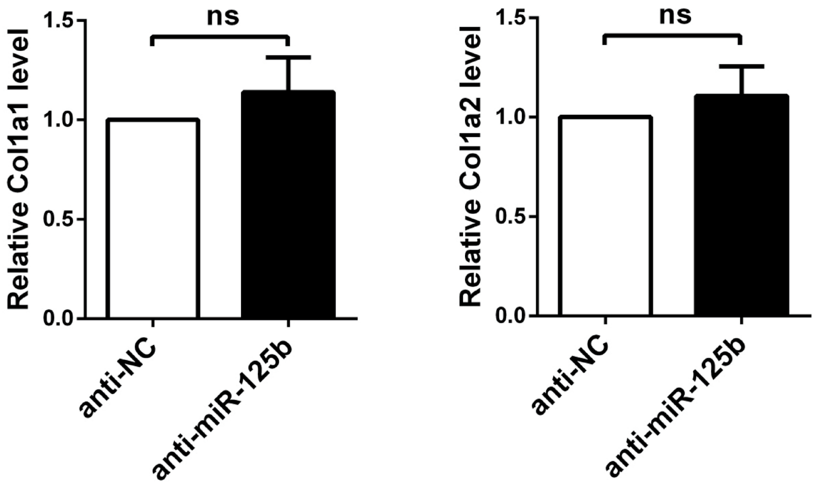

Figure S7. Knockdown of miR-125b did not affect the basal levels of *Col1a1* and *Col1a2*. JS1 cells were transfected with anti-NC or anti-miR-125b for 48 hours, then the mRNA levels of *Col1a1* and *Col1a2* were analyzed by qRT-PCR. Data are presented as mean±SEM. ns, not significant.

Figure S8

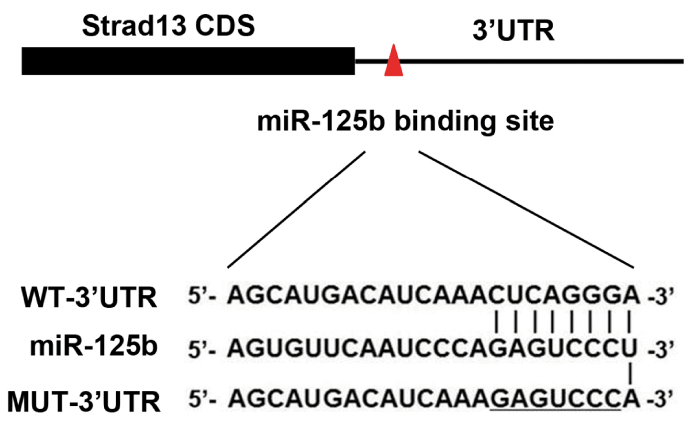

Figure S8. The schematic diagram of the luciferase reporter plasmids that contain either wild-type or mutant 3'UTR of *Stard13*. The segments containing miR-125b binding site were cloned into psiCHECK-2 vector.

Figure S9

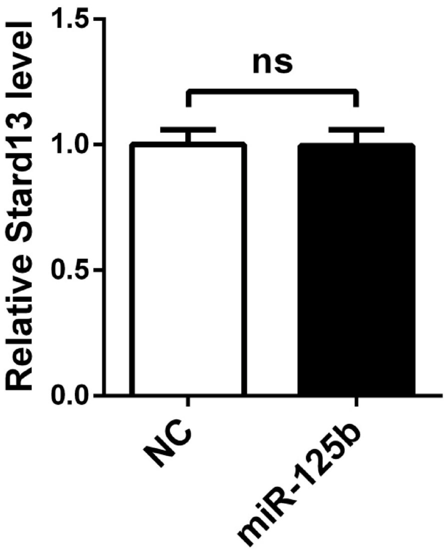

Figure S9. Overexpression of miR-125b did not affect the mRNA level of *Stard13*. JS1 cells were transfected with NC or miR-125b mimics for 48 hours, then the mRNA level of *Stard13* was analyzed by qRT-PCR. Data are presented as mean±SEM. ns, not significant.

Figure S10

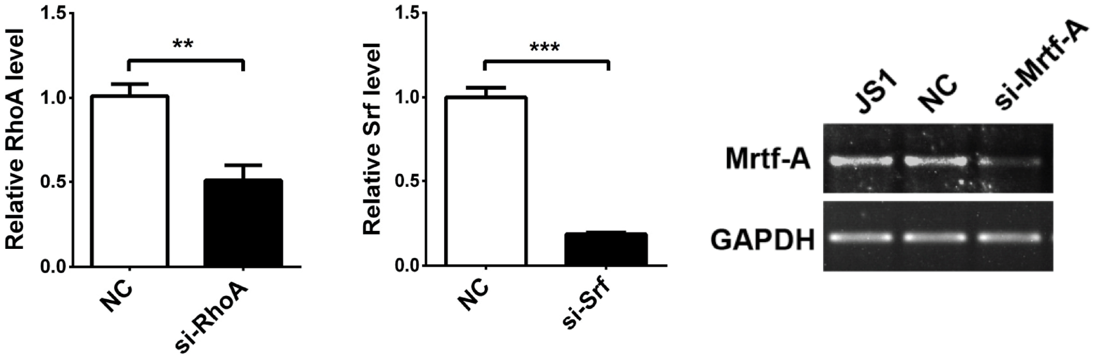

Figure S10: The knockdown effect of siRNAs for *RhoA, Srf* and *Mrtf-A*. JS1 cells were transfected with the indicated RNA for 48 hours and then subjected to qRT-PCR or RT-PCR analysis. Data are presented as mean±SEM. **, *P < 0.0*5; ***, *P < 0.0*01.

Supplemental Tables

Table S1. Characteristics of study subjects

Case	Gender	Age(years)	ALT(U/L)[1]	AST(U/L)[1]	TBIL(μmol/L)[1]	HBsAg[2]	Cirrhosis[3]
**Liver fibrosis**							
1	Male	60	741	1590	19.1	+	+
2	Male	36	39	29	10.8	+	+
3	Male	57	51	60	27.9	+	+
4	Male	60	46	45	20.9	+	+
5	Male	40	39	62	17	+	+
6	Male	61	163	104	770.1	+	+
7	Male	41	48	26	61	+	+
8	Male	38	19	10	48.4	-	+
9	Male	46	69	36	71.5	+	+
**Hepatic hemangiomas**							
1	Male	50	16.6	17.8	19	+	-
2	Female	56	11.9	16	12.7	-	-
3	Male	51	39.3	73.1	16.1	+	-
4	Female	42	34	44.6	9.6	+	-
5	Female	50	14.9	17.1	10.3	+	-
6	Female	53	16.2	16.4	10.8	-	-
7	Male	41	46	28.9	15.8	-	-
8	Male	49	17	19.3	13.3	-	-
9	Female	46	11.5	13.3	3.6	+	-

[1] ALT, alanine aminotransferase; AST, aspartate aminotransferase; TBIL, total bilirubin;

[2] HBsAg, hepatitis B surface antigen; detected by ELISA; "+", presence; "-", absence.

[3] Determined by pathological examination; "+", presence; "-", absence.

Table S2. Sequences of RNA and DNA Oligonucleotides

Name	Sense Strand (5'-3')	Antisense Strand (5'-3')
**miRNA and siRNA duplexes**		
miR-125b	UCCCUGAGACCCUAACUUGUGA	ACAAGUUAGGGUCUCAGGGCUU
si-Stard13-1	UGCUCUAUUCACAGCGAAAdTdT	UUUCGCUGUGAAUAGAGCAdGdA
si-Stard13-2	CAGACCAAACAGACGACUAdTdT	UAGUCGUCUGUUUGGUCUGdGdT
si-Stard13-3	GCUCCUGACUACCGAGACAdTdT	UGUCUCGGUAGUCAGGAGCdTdT
si-RhoA-1	GUGAUGGAGCUUGUGGUAAdTdT	UUACCACAAGCUCCAUCACdCdA
si-RhoA-2	CGGAAUGACGAGCACACGAdTdT	UCGUGUGCUCGUCAUUCCGdAdA
si-Srf-1	CCAACGGGACUGUGCUCAAdTdT	UUGAGCACAGUCCCGUUGGdCdA
si-Srf-2	CCUACCAGGUGUCGGAAUCdTdT	GAUUCCGACACCUGGUAGGdTdG
si-Tgfbr1-1	ACAAGAAAGCAUUGGCAAAdTdT	UUUGCCAAUGCUUUCUUGUdAdA
si-Tgfbr1-2	CUGACAGCUUUGCGAAUUAdTdT	UAAUUCGCAAAGCUGUCAGdCdC
si-Mrtf-A-1	CAAGUCUGCCAGCGAGAAAdTdT	UUUCUCGCUGGCAGACUUGdGdG
si-Mrtf-A-2	AGGACUAUUUGAAACGGAAdTdT	UUCCGUUUCAAAUAGUCCUdCdG
si-Smad2-1	GCUGAGUGCCUAAGUGAUAdTdT	UAUCACUUAGGCACUCAGCdAdA
si-Smad2-2	CAUCAUGAGCUCAAGGCAAdTdT	UUGCCUUGAGCUCAUGAUGdAdC
si-Smad3-1	CCGUAUGAGCUUCGUCAAAdTdT	UUUGACGAAGCUCAUACGGdAdT
si-Smad3-2	UGUCCAAUGUCAACCGGAAdTdT	UUCCGGUUGACAUUGGACAdGdT
NC	UUGUACUACACAAAAGUACUG	GUACUUUUGUGUAGUACAGUU
**miRNA inhibitors**		
anti-NC	GUGGAUAUUGUUGCCAUCA	
anti-miR-125b	UCACAAGUUAGGGUCUCAGGGA	
**Primers for RT-PCR or qRT-PCR**		
α-SMA	ATGCAGAAGGAGATCACAGC	CAGCTTCGTCGTATTCCTGT
Col1a1	ATGATGCTAACGTGGTTCGT	TGGTTAGGGTCGATCCAGTA
Col1a2	GGAGGGAACGGTCCACGAT	GAGTCCGCGTATCCACAA
Timp-1	GCAACTCGGACCTGGTCATAA	CGGCCCGTGATGAGAAACT
Fn1	ATGAGCGCCCTAAAGATTCC	CCAGCAGCATGATCAAAACA
Stard13	CCACTGGGGTCAGAGAAAGA	ATGATGGCCGTAAGACGAAG
RhoA	CTTCAGCAAGGACCAGTTCCCA	GGCGGTCATAATCTTCCTGTCC
Srf	ATGAGTGCCACTGGCTTTG	CTGTGAATGCTGGCTTCAGT
Tgfbr1	GGTCTTGCCCATCTTCACAT	TGGCAGAATCATGTCTCACAG
Mrtf-A	CCAGGACCGAGGACTATTTG	TGTGCAATCTTTTCATTGAGG
Smad2	ACTACACCCACTCCATTCCA	GCGATTGAACACCAGAATGC
Smad3	TTTTCGTCCAGTCTCCCAAC	CTGGTCACTGTCTGTCTCCT
GAPDH	AACTTTGGCATTGTGGAAGG	CACATTGGGGGTAGGAACAC
**Primers for cloning (Restriction enzyme sites were underlined)**		
Stard13-3'UTR-WT	AGTCTCGAGGCTCAGGACCTGGAAGACTG	AGTGCGGCCGCGGAAGTGCCATGTGAGGAGA
Stard13-3'UTR-MUT	CAGCATGACATCAAAGAGTCCCAAGAAGAAGGGAAAG	CTTTCCCTTCTTCTTGGGACTCTTTGATGTCATGCTG
pCDH-Ctrl	AGTGAATTCATGGTGAGCAAGGGCGAGGAG	AGTGGATCCTTACTTGTACAGCTCGTCCATGCC
pCDH-miR-125b sponge	AGTGAATTCATGGTGAGCAAGGGCGAGGAG	AGTGGATCCAGAGTGGAGTCGTCGACGCC
